# Antimicrobial activity, membrane interaction and structural features of short arginine-rich antimicrobial peptides

**DOI:** 10.3389/fmicb.2023.1244325

**Published:** 2023-10-05

**Authors:** Bruna Agrillo, Alessandra Porritiello, Lorena Gratino, Marco Balestrieri, Yolande Therese Proroga, Andrea Mancusi, Loredana Cozzi, Teresa Vicenza, Principia Dardano, Bruno Miranda, Pablo V. Escribá, Marta Gogliettino, Gianna Palmieri

**Affiliations:** ^1^Ampure S.R.L., Napoli, Italy; ^2^National Research Council (IBBR-CNR), Institute of Biosciences and Bioresources, Napoli, Italy; ^3^Department of Food Microbiology, Istituto Zooprofilattico Sperimentale del Mezzogiorno, Portici, Italy; ^4^Department of Food Safety, Nutrition and Veterinary Public Health, Istituto Superiore di Sanità, Rome, Italy; ^5^National Research Council (ISASI-CNR), Institute of Applied Sciences and Intelligent Systems, Napoli, Italy; ^6^Laboratory of Molecular Cell Biomedicine, University of the Balearic Islands, Palma, Spain; ^7^Laminar Pharmaceuticals, Palma, Spain; ^8^Materias S.R.L., Naples, Italy

**Keywords:** antimicrobial compound, cationic arginine-rich peptide, arginine, membrane interaction, spectroscopy

## Abstract

Antimicrobial activity of many AMPs can be improved by lysine-to-arginine substitution due to a more favourable interaction of arginine guanidinium moiety with bacterial membranes. In a previous work, the structural and functional characterization of an amphipathic antimicrobial peptide named RiLK1, including lysine and arginine as the positively charged amino acids in its sequence, was reported. Specifically, RiLK1 retained its β-sheet structure under a wide range of environmental conditions (temperature, pH, and ionic strength), and exhibited bactericidal activity against Gram-positive and Gram-negative bacteria and fungal pathogens with no evidence of toxicity on mammalian cells. To further elucidate the influence of a lysine-to-arginine replacement on RiLK1 conformational properties, antimicrobial activity and peptide-liposome interaction, a new RiLK1-derivative, named RiLK3, in which the lysine is replaced with an arginine residue, was projected and characterised in comparison with its parental compound. The results evidenced that lysine-to-arginine mutation not only did not assure an improvement in the antimicrobial potency of RiLK1 in terms of bactericidal, virucidal and fungicidal activities, but rather it was completely abolished against the hepatitis A virus. Therefore, RiLK1 exhibited a wide range of antimicrobial activity like other cationic peptides, although the exact mechanisms of action are not completely understood. Moreover, tryptophan fluorescence measurements confirmed that RiLK3 bound to negatively charged lipid vesicles with an affinity lower than that of RiLK1, although no substantial differences from the structural and self-assembled point of view were evidenced. Therefore, our findings imply that antimicrobial efficacy and selectivity are affected by several complex and interrelated factors related to substitution of lysine with arginine, such as their relative proportion and position. In this context, this study could provide a better rationalisation for the optimization of antimicrobial peptide sequences, paving the way for the development of novel AMPs with broad applications.

## 1. Introduction

Antimicrobial peptides (AMPs), also defined as small molecular-mass proteins, are short amino acid sequences, able to efficiently kill or prevent the growth of microorganisms through various and broad mechanisms of action. These compounds have been found in different forms of life from microorganisms to humans including fish, plants, amphibians, invertebrates, birds, and mammals. Moreover, AMPs are evolutionarily conserved in the genome and in the higher organisms they are essential components of the innate and adaptive immune systems, playing an essential role in defending against microbial infections (Giuliani et al., 2008; Peters et al., 2010; Wang S. et al., 2016; Huan et al., 2020; Erdem Büyükkiraz and Kesmen, 2022).

In 1939, the microbiologist René Dubos isolated from a soil Bacillus strain the first antimicrobial compound, named gramicidin, which was able to protect mice from pneumococcal infection (Van Epps, 2006). Next, several AMPs have been identified and characterised from both the prokaryotic (bacteriocins) and eukaryotic (cathelicidins, defensins) organisms, allowing to collect several information on their most basic chemical-physical parameters (Zasloff, 2002; Phoenix et al., 2013; Yazici et al., 2018). These studies provided important indications for a further development of these compounds, leading to *in silico* design of new antimicrobial compounds on the basis of natural or non-natural peptide sequences. In general, AMPs share several key properties, including amphipathicity, mean hydrophobicity, and net cationic charge owed to the presence of lysine and arginine as basic residues. Moreover, these compounds can adopt a variety of conformational structures, such as β-sheet and random-coil structures, although most of them exhibit α-helical structures (Ebenhan et al., 2014; Wang et al., 2019; Pirtskhalava et al., 2021).

AMPs explicate their antimicrobial activity through many different mechanisms. The most studied AMPs initially interact with common targets on surface of cells without the need for specific receptors. The amphipathicity and the net cationic nature of these membrane-active AMPs are believed to be the main driving force for their cell selectivity and interaction resulting in membrane integrity disruption (Pirtskhalava et al., 2021). Indeed, cationic AMPs can strongly bind the negatively charged bacterial membranes, due to the presence of a large proportion of phospholipids (such as phosphatidylglycerol and cardiolipin) and peptidoglycans (such as lipoteichoic acids) in the Gram-positive bacteria, and lipopolysaccharides (LPS) in the outer membrane for Gram-negative bacteria (Andersson et al., 2016; Omardien et al., 2016). On the other hand, the architecture of bacterial membranes markedly differs from that of the mammalian cell envelope which includes mostly zwitterionic phospholipids (such as sphingomyelin and phosphatidylcholine), providing a reasonable means for positively charged AMPs to target bacteria in a selective manner. In this context, drugs and biological agents targeting membrane lipid bilayers has received great attention during the latest years (Escribá et al., 2015). Thus, “membrane lipid therapy” (or melitherapy) arises as a new approach to developed therapeutic agents to treat different conditions, including infectious diseases.

Therefore, the strategy for the development of an efficient AMP includes a good balance among charge, hydrophobicity, amphipathicity, secondary and tertiary structure, and mode of action which are all important variables to identify candidates of success However, the discovery in the last decade of a lot of AMPs with great diversity in the distribution of amino acids along their sequence, made the study of these peptides more complex. In contrast to the difficulty intrinsic to this variety, short amphipathic peptides can represent simpler models of AMPs to investigate the role of hydrophobic and electrostatic interactions in peptide structure-activity correlations (Torres et al., 2019; Pirtskhalava et al., 2021; Chakraborty et al., 2022).

Recently, a new 10-amino acid peptide, namely RiLK1 (Agrillo et al., 2020; Falcigno et al., 2021), was designed based on the dodecapeptide 1018-K6 (Palmieri et al., 2018; Colagiorgi et al., 2020; Festa et al., 2021; Ambrosio et al., 2022), a compound derived from a bovine HDP (host defence peptide) bactenecin, belonging to the cathelicidins family. Structural and functional analysis, revealed that RiLK1 is extremely active toward fungi, viruses, Gram-positive and-negative bacteria at low micromolar concentrations, showing no effects on human cell lines investigated in terms of viability and morphology. Moreover, results evidenced a conformational propensity of RiLK1 to self-assembling in regular structures in a more efficient way than the parent peptide 1018-K6, providing a possible explanation for the potent bactericidal, antifungal and anti-biofilm activities exhibited by RiLK1 in comparison to 1018-K6.

Herein, with the aim to investigate the effects of an increased arginine (R) composition in the sequence of RiLK1 (mixed R/K), a single amino acid substitution of the basic lysine (K) residue with arginine was projected. Indeed, despite the identical charge of R and K, the former residues are more prevalent in naturally occurring AMPs than latter, suggesting that the guanidinium group may be preferable for antimicrobial activity than the amine group, as confirmed in many studies (Yeaman and Yount, 2003; Hristova and Wimley, 2011).

Therefore, the new peptide, named RiLK3 (all R), was projected and characterised in comparison with its parental compound RiLK1 in terms of structural, functional and self-assembly properties as well as peptide-membrane interaction. The changes in the conformational propensity of peptides were measured by CD and fluorescence spectroscopy while their tendency for self-assembling was analysed by Fourier transform infrared spectroscopy (FT-IR) and cross-linking experiments. Then, the antimicrobial activity against the pathogen’s bacteria, fungi and viruses was evaluated together with peptide-lipid membrane interactions.

## 2. Materials and methods

### 2.1. Synthesis and *in silico* design of RiLK3

The peptides RiLK1 and RiLK3 used in this work were purchased from GenScript Biotech (Leiden, Netherlands) and obtained at >95% purity. The peptides were stored as a lyophilized powder at −20°C. Before experimental analyses, fresh solutions in 100% dimethyl sulfoxide (DMSO, Sigma Aldrich, Milan, Italy) were prepared, briefly vortexed, and sonicated, and these samples were used as stock solutions. The following web server and software were utilized to determine all the relevant physicochemical properties of RiLK1 and RiLK3: PlifePred (PPred) (Mathur et al., 2018), PEPlife (Mathur et al., 2016), Antimicrobial Peptide Database3 (APD3) (Wang G. et al., 2016), and collection of antimicrobial peptides (CAMP) (Waghu et al., 2016).

### 2.2. Antibacterial assay

The minimum bactericidal concentration (MBC) was determined by the standard broth micro-dilution method in accordance with the Clinical and Laboratory Standards Institute (2015). For micro-broth dilution assay, *Listeria monocytogenes* (isolated from food products), *Escherichia coli* (strain ATCC 25922), *Staphylococcus aureus* (strain ATCC 25923), *Salmonella Typhimurium* isolated from food and *Pseudomonas aeruginosa* (strain ATCC 27853) were grown in BPW (Thermo Fisher, Milan, Italy). Bacterial cells were cultured at 37°C in the culture media until collection and then diluted in fresh broth to a final concentration of 1.5 × 10^3^ CFU/mL (CFU, colony forming units). Next, serial dilutions of RiLK3 and RiLK1 in BPW (ranging from 1 to 75 μM), prepared starting from stock solutions in DMSO, were added to each bacterial suspension and incubated at 37°C for 6 h. Control samples containing only cell suspension and DMSO were also used. The MBCs were determined by transferring 50 μL of each bacterial cell suspensions onto selective agar plates (*L. monocytogenes*, Agar Listeria acc. to Ottaviani & Agosti (ALOA)—Microbiol, Macchiareddu (CA)—Italy; *S. typhimurium*, *Salmonella* Chromogenic agar—Oxoid, Madrid, Spain; *S. aureus*, Baird Parker agar base—Oxoid, Madrid, Spain; β-glucuronidase-positive *E. coli*, Triptone Bile X-glucuronide Agar (TBX)—Oxoid, Madrid, Spain; *P. aeruginosa*, *pseudomonas* agar base with CFC supplement-Oxoid, Madrid, Spain) incubated 24/48 h at 37°C for *L. monocytogenes* (ISO 11290-1:2017), *S. typhimurium* (ISO 6579-1:2020), and *S. aureus* (ISO 6888-1:1999) while *E. coli* (ISO 16649-1:2018) was incubated 24 h at 44°C and *P. aeruginosa* at 25°C for 48 h (ISO 13720:2010). MBC is defined as the lowest concentration of peptide at which more than 99.9% of the bacterial cells are killed. All values were obtained as the mean of three independent experiments conducted in triplicate.

### 2.3. Antifungal assays

Fungal strains were purchased from the American Type Culture Collection (ATCC, Manassas, VA, United States) as follows: *Aspergillus brasiliensis* ATCC 9341 and *Candida albicans* ATCC 14053 strains. Briefly, the cell suspension of both fungal species was adjusted to 1.0 × 10^5^ CFU/mL in buffered peptone water (BPW) (bioMerieux, Florence, Italy). Peptide stock solutions in DMSO were added to the cell suspension at a final concentration of 25 μM and 50 μM and incubated for 6 h a 37°C. The minimum fungicidal concentration (MFC) was determined by plating 100 μL cultures on DG18 plates (Dichloran 18% Glycerol Agar—ISO 21527-2) for CFU counting. After 7 days at 25°C, the MFC was defined as the lowest peptide concentration that resulted in 99.9% killing compared with the drug-free group. The analyses were performed in triplicate on three different experiments.

### 2.4. Antiviral assay

The hepatitis A virus (HAV) HM 175 strain was replicated in Frp3 cells cultured in minimum essential medium with Earle’s salts (MEM), supplemented with 1% glutamine, 2% non-essential amino acids and 2% fetal bovine serum (FBS), at 37°C in 5% CO_2_. EuroClone, (Milan, Italy) provided all cell culture media. The viral suspension was prepared by 3 cycles freeze and thaw lysis of infected monolayer, clarified using low-speed centrifugation (800 × g) to remove residual debris, then the sample was aliquoted and stored at −80°C until use. The obtained HAV stock suspension revealed a final concentration of 4.6 × 10^6^ TCID50/mL, calculated by determining the 50% tissue culture infectious dose by the Reed and Muench (1938) method using tenfold serial dilutions in 24-well plates. Preliminary tests were performed on the Frp3 cell lines (non-human primate cell line derived from Fetal kidney) to determine the peptide concentration that did not produce any cytotoxic effects. Cell viability was evaluated by 3-(4,5 dimethilthiazol-2-yl)-2,5-dipheniltetrazolium bromide (MTT) assay (Manger et al., 1993). Peptide solutions were prepared in serum-free MEM at several concentrations in the range from 100 to 10 μM, treated overnight at 4°C with antibiotic/anti-mycotic solution (EuroClone) and assayed on 24–48 h cell monolayers in a 24-well plate. The monolayers were incubated for 1 h at 37°C in 5% CO_2_. Thereafter, cells were washed twice with Dulbecco’s Phosphate Buffer Solution (DPBS, EuroClone) and maintained with MEM supplemented with 2% of FBS for 48 h in 5% CO_2_ at 37°C. After that, the medium was removed and 300 μL of MTT (Sigma Aldrich, Milan, Italy) solution (5 mg/mL) was added. The monolayers were incubated for 15–30 min at 37°C in 5% CO_2_. Therefore, the MTT was removed and 500 μL DMSO was added to each well to dissolve the purple formazan. The absorbance was measured at 570 nm. DMSO and culture medium were used as controls. Therefore, solutions of 40 μM and 80 μM concentration for each peptide were chosen to treat HAV at a concentration of 4.6 × 10^4^ TCID50/mL. The suspensions containing the peptides and virus were incubated for 1 h at room temperature (RT). Then the viral infectivity was investigated on Frp3 cells. Untreated HAV suspension and 40 μM and 80 μM peptide solutions, incubated at the same conditions, were used as controls. Each treatment was assayed in triplicate. Viral titrations were performed by determining the TCID50/mL. Briefly, 100 μL of serial tenfold dilutions of each sample were assayed in 24-well tissue culture plates containing 24–48 h monolayers of Frp3 cells, and incubated for 1 h in 5% CO_2_ at 37°C. After that, the wells were washed twice with 200 μL of PBS, and 500 μL of MEM supplemented with 2% of FBS were added to each well. The infections were carried out for up to 14 days in 5% CO_2_ at 37°C with a daily visual inspection. After 7 days the culture medium was changed. The virucidal efficacy of peptides was estimated by comparing the titres of the viral suspension treated with the titre of the untreated virus. The reduction in viral infectivity was evaluated as log reduction value (LRV) by calculating the log10 N0/N1, where N0 is the titre for untreated viral suspension and N1 is the titre for treated viral suspension.

### 2.5. Circular dichroism spectroscopy

Circular dichroism (CD) analysis was performed by Jasco J-810 spectropolarimeter. The samples were loaded into a quartz cuvette of 0.1 cm path length (Hellma Analytics) and the spectra were recorded in the 195 nm–250 nm range at a scan speed of 20 nm/min, by averaging 5 scans and in the presence or absence of SDS. The effect of pH on the secondary structure of RiLK1 and RiLK3 was analysed by dissolving the samples at a concentration of 80 μM in different buffer solutions at 10 mM concentration: glycine-HCl, pH 2.0; Tris-HCl, pH 7.0; glycine-NaOH, pH 11.0. Next, SDS (50 mM final concentration) was added to each sample, which was incubated up to 48 h at 25°C and analysed by CD spectroscopy. The folding kinetic measurements of the peptides were performed after the addition of SDS (50 mM) to each sample (80 μM in 10 mM Tris-HCl, pH 7.0) up to 24 h incubation. CD experiments were also carried out in 10 mM Tris-HCl buffer pH 7.0 as function of SDS concentration at a peptide concentration of 80 μM. For thermal stability analyses, the peptides were prepared to a final concentration of 80 μM in 10 mM Tris-HCl, pH 7.0 in the presence of 50 mM SDS and then they were incubated at 4, 37 and 90°C up to 48 h before acquired the CD spectra. A blank spectrum of a sample containing all components except the peptide was acquired for the baseline-correction of the CD spectra of the peptide. The mean residue ellipticity ([*θ*], deg. cm^2^ dmol^−1^) was obtained by the equation [*θ*] = 100 θ/*cnl*, where *θ* is the ellipticity (mdeg), *c* is the peptide concentration (mM), *n* is the number of residues, and *l* is the path length (cm). The percentage of secondary structure was estimated by the DICHROWEB site (Whitmore and Wallace, 2004, 2008), using the K2D algorithm (Perez-Iratxeta and Andrade-Navarro, 2008).

### 2.6. Fluorescence spectroscopy

Trp fluorescence emission spectra were recorded at 25°C on a Shimadzu RF-6000 spectrofluorometer (Kyoto, Japan) with both excitation and emission slit widths set at 5 nm. The intrinsic tryptophan was excited at a wavelength of 280 nm and the emission was monitored between 300 and 400 nm. The folding kinetic experiments of RiLK1 and RiLK3 were performed after the addition of SDS (50 mM) to each sample (80 μM concentration in 10 mM Tris-HCl buffer pH 7.0) up to 24 h incubation. Fluorescence measurements were also carried out in 10 mM Tris-HCl buffer pH 7.0 as a function of SDS concentration at a peptide concentration of 80 μM. The effect of pH on peptide folding was analysed by dissolving the peptides at a final concentration of 50 μM in different buffer solutions at 10 mM concentration: glycine-HCl, pH 2.0; Tris-HCl, pH 7.0; glycine-NaOH, pH 11.0. Then, SDS (50 mM) was added to each sample, which was incubated up to 48 h at 25°C and monitored by fluorescence spectroscopy. For thermal stability, the peptides were prepared to a final concentration of 80 μM in 10 mM Tris-HCl buffer pH 7.0 in the presence of 50 mM SDS and then they were incubated at 4, 37 and 90°C up to 48 h.

### 2.7. Peptide cross-linking

Either RiLK3 or RiLK1 at 240 μM concentration in the presence of SDS micelles (150 mM) in 10 mM sodium phosphate buffer pH 7.0 was cross-linked with or without glutaraldehyde at 4% (v/v) in the dark at 37°C for 24 h. The samples (10 μL) were analysed by tris-tricine SDS-PAGE (17%). The electrophoresis was conducted at 30 mA for 1 h at room temperature. Gel filtration chromatography was performed on Superdex 30 Increase (10/300 GL, Pharmacia Biotech, Milan, Italy) column connected to an AKTA FPLC system (GE Healthcare, Italy), pre-equilibrated with 50 mM Tris-HCl buffer (pH 7.5) containing 150 mM NaCl and 20% Acetonitrile. Standard protein markers (BioRad code 151-1901) were utilized to calibrate the gel filtration column. The fractions eluted by the column were collected and analysed by fluorescence spectroscopy.

### 2.8. Fourier transform infrared spectroscopy analysis

A Nicolet iS50 Fourier transform infrared (FT-IR) spectrometer (Thermo Scientific) equipped with macro-diamond based attenuated total reflection (ATR) module (smart iTX-diamond by Thermo Scientific) and DTGS KBr detector was utilized to collect FT-IR spectra of sample powders. Powders were in direct contact with the diamond plate of the ATR module using a pressuring tip. The background was acquired from the diamond plate in the air without samples. All spectra were recorded using 16 scans in the range from 4000 to 525 cm^−1^ with a 0.482 cm^−1^ spectral resolution. Each sample was analysed in triplicate and averaged. Moreover, to determine any secondary structure of samples, according to Wi et al. (1998), Rea et al. (2014) and Portaccio et al. (2015), the amide I band was analysed. In particular, the deconvolution of FT-IR spectra in the range 1700÷1600 cm-1 was released by fitting data with multi-Lorenztian peaks corresponding to the *minima* of second derivative spectra. Second derivative spectra were obtained with Savitsky–Golay derivative function algorithm on 7 data points by in-home software.

### 2.9. Preparation of model membranes: multilamellar vesicles

Model membranes (liposomes) were prepared using lipid stock solutions (10 mM) in chloroform:methanol (2:1, v/v) of 1-palmitoyl-2-oleoyl-sn-glycero-3-phosphoethanolamine (POPE) (Avanti Polar Lipids, Alabaster, United States), 1,2-dioleoyl-sn-glycero-3-phosphoethanolamine (DOPE) (Avanti Polar Lipids, Alabaster, United States), L-α-lysophosphatidylcholine (Egg Lecithin, PC) (Avanti Polar Lipids, Alabaster, United States), N-Acyl-D-sphingosine-1-phosphocholine (chicken egg yolk, SM) (Sigma-Aldrich, St. Louis, MO, United States), phosphatidyl serine (PS) (Avanti Polar Lipids, Alabaster, United States), 1′,3′-bis [1,2-dioleoyl-sn-glycero-3-phospho]-glycerol (Cardiolipin 18:1, CL) (Avanti Polar Lipids, Alabaster, United States) and 1-palmitoyl-2-oleoyl-sn-glycero-3-phosphatidylglycerol (POPG) (Larodan AB, Sweden). Lipid films were made in glass vials by mixing a volume of phospholipid stock solutions to achieve the desired molar ratio as reported below. Next, the solutions (2 mL) were dried under argon flow and then subjected to a vacuum for at least 3 h to remove traces of the solvent. A sufficient volume of binding buffer composed of 4-(2-hydroxyethyl) piperazine-1-ethanesulfonic acid 10 mM (HEPES, Sigma-Aldrich) and NaCl 100 mM (Sigma-Aldrich), pH 7.2 was used to resuspend the lipidic film, yielding 2 mM lipid phosphorus. Lipid suspensions were freshly prepared before each experiment. The membrane lipid composition for eukaryotic and prokaryotic cells (% mol) was reported in Table 1.

**Table 1:** Lipid composition of eukaryotic and prokaryotic membranes.

Membrane	PC	SM	PS	DOPE	POPE	POPG	CL
Eukaryotic membrane^a^	40	15	5		40		
Zwitterionic membrane	100						
*Salmonella Typhimurium* ^b^ ^,GN^				78		18	4
*Staphylococcus aureus* ^c^ ^,GP^						58	42

PC, phosphatidylcholine; SM, sphingomyelin; PS, phosphatidylserine; DOPE, dioleoyl-phosphatidylethanolamine; POPE, palmito-yl-oleoyl-phosphatidylethanolamine; POPG, palmitoyl-oleoyl-phosphatidylglycerol; CL, cardiolipin. GN, Gram-negative bacteria; GP, Gram-positive bacteria.

a Casares et al. (2019).

b Barbosa et al. (2019).

c Epand and Epand (2011).

### 2.10. Lipid binding assay

Reaction mixtures were prepared in Eppendorf tubes combining each lipid solution (at the fixed lipid phosphorus molar concentration of 1800 μM) with pre-formed liposomes containing different amounts of peptide, ranging from 20 to 100 μM (when saturation did not occur, higher peptide concentrations were tested). The samples were vigorously vortexed and incubated at room temperature for 30 min to allow binding. Analogous samples, made with the buffer solution vehicle rather than the stock peptide solution, were used as negative controls. At the end of the incubation period, the solutions were transferred carefully to polycarbonate centrifuge tubes (1 mL, 8 × 51 mm: Beckman Coulter, United States) and centrifuged at 60,000× g (20,000 rpm, outer row) for 1 h at 20°C (Beckman LE-80 Ultracentrifuge; rotor type 25). Therefore, the supernatant was removed and the pellet was washed (two-three times) with the binding buffer to eliminate the aggregated peptide eventually precipitated and resuspended with the same buffer containing sodium dodecyl sulfate (SDS, Thermo Fisher, Germany) at a final concentration of 1%. The binding of the peptide to the model membranes was assessed by quantifying the amount of RiLK1 or RiLK3 in the pellet and supernatant using calibration curves generated by adding known amounts of the peptide to control supernatants or pellets of vesicles prepared in the absence of the peptide (see above). Peptide binding to multilamellar vesicles (MLVs) was monitored by fluorescence spectroscopy taking advantage of the presence of the tryptophan residues in RiLK1 or RiLK3.

### 2.11. Steady-state tryptophan fluorescence

Tryptophan (Trp) fluorescence spectra were recorded for each supernatant and pellet sample after 30 min of stirring at 900 rpm in a Thermo–Shaker (TS-100: Biosan) at room temperature (Ambrosio et al., 2022). The variation in Trp emission was monitored between 300 and 450 nm excitation, at *λ*_ex_ = 280 nm, by a Shimadzu RF-6000 spectrofluorometer (Kyoto, Japan). Slit widths were 2.5 nm for excitation and 5 nm for emission, and each spectrum was corrected by subtracting the liposome background.

### 2.12. Morphological characterization and surface charge measurements

The hydrodynamic size and ζ-potential of *Salmonella-and Staphylococcus*-like liposomes were measured by Zetasizer Nano-ZS instrument (Malvern Instrument Ltd., Cambridge, United Kingdom) equipped with a He–Ne laser (633 nm, fixed scattering angle of 173°, 25°C). The size (*d*) and the polydispersity index (PDI) of the obtained liposomes (at an initial concentration of 2 mM) were measured by diluting them down to 0.2 mM in MilliQ water. The liposome suspensions (1 mL for each type) were inserted in a standard disposable cuvette and three measurements (*n* = 3) of their size and PDI were performed. The ζ-potential of the bacterial-mimic liposomes and the peptides (before and after their interaction with them) were measured in triplicate (*n* = 3) by using disposable zeta-potential cuvettes (1 mL). ζ-potential measurements were performed for peptides (RiLK 1 and RiLK 3) at a concentration of 0.01 mM, and after liposome:peptide interaction at two different ratios (20:1 and 2:1). The interactions between bacterial-like liposomes and peptides were performed as reported in Section 2.10. To achieve the suitable concentrations for ζ-potential measurements, the incubations were performed at 10 times higher concentrations and underwent a 1:10 dilution in MilliQ water before ζ-potential measurements. The two different liposome:peptide ratios were obtained by fixing the peptide concentration at 0.1 mM and changing the liposome concentration, accordingly.

### 2.13. Statistical analyses

Lipid binding and antimicrobial assays were performed by GraphPad Prism^®^ (version 9.5.0, software San Diego, CA, United States). All experiments were carried out at least three times and the data were reported as the mean (M) ± standard deviation (s.d.). The statistical significance of differences between samples in the presence or absence of peptides was calculated through one-way analysis of variance (ANOVA) with Bonferroni *post hoc* comparisons, with a significance level of *p* < 0.05.

## 3. Results and discussion

### 3.1. Rational design of RiLK3

The rational design of AMPs represents a practical strategy to obtain a peptide with improved antibacterial properties. Therefore, a study for analysing the structural elements which govern the antimicrobial action of the already characterised peptide RiLK1 was performed with the aim to provide important information for further modifications of key residues and the generation of new RiLK1-based antimicrobial agents.

It is widely reported that cationic residues are fundamental for the antimicrobial activity of amphipathic AMPs as they attract the peptide to the negatively charged bacterial membranes via electrostatic interaction (Yeaman and Yount, 2003). In this context, arginine is reported to be more efficacious in mediating peptide-membrane interactions as its guanidinium moiety displays a stronger H-bonding capability with the phospho-rich membrane surface of the bacteria compared to the primary amine moiety of lysine (Yeaman and Yount, 2003; Hristova and Wimley, 2011). Moreover, the arginine side-chains can form bidentate interactions with lipids leading to enhance the membrane curvature and then the activity, while lysine residues do not induce this curvature on their own, producing only monodentate interactions (Schmidt et al., 2012; Cutrona et al., 2015). Afterwards, increased arginine/guanidinium vs. lysine/amine composition appears to improve the antimicrobial potency of peptides, although this trend is not universally observed (Cutrona et al., 2015).Therefore, to investigate on the structure and function of RiLK1, a 10-mer analogue, named RiLK3 was projected by replacing the lysine residue at position 3 with arginine (Hristova and Wimley, 2011). Following this *in silico* site-directed mutagenesis approach, the designed AMP was characterised using four online software packages to predict the relevant physicochemical parameters, which are recognized to be necessary to perform the biological function. This investigation was carried out to make sure that RiLK3 retained similar or better features as the parental ones. According to the data reported in Table 2, the RiLK1-derivative peptide displayed improved performances compared to the RiLK1 in terms of Boman index, hydropathicity and hydrophobicity, which are properties considered important for peptide-membrane interactions (Torres et al., 2017).

**Table 2:** Physicochemical properties of the mutated peptide RiLK3 in comparison with those of the parent RiLK1.

Parameters	RiLK1^a^	RiLK3
Sequence	NH_2_-RLKWVRIWRR-CONH_2_	NH_2_-RLRWVRIWRR-CONH_2_
Molecular weight (Da)	1468.96	1495.83
Bomax index (kcal/mol)	4.70	5.6
Net charge	+5	+5
Half-life (sec)	855.71	852.61
Hydrophobicity	−0.56	−0.63
Hydropathicity	−1.12	−1.18
Amphipathicity	1.35	1.23
Hydrophilicity	0.31	0.31

The mutated amino acid residue of the parental peptide RiLK1 is highlighted in red.

a Agrillo et al. (2020).

### 3.2. Antibacterial, antifungal and antiviral activity of RiLK3

To assess the impact of the performed substitution on the antibacterial activity of the mutant RiLK3, the minimal bactericidal concentrations (MBCs) of the peptide were measured against some of the most representative Gram-negative (*S. typhimurium*, *E. coli* and *P. aeruginosa*) and Gram-positive foodborne pathogens (*S. aureus* and *L. monocytogenes*), and compared with those of the parental RiLK1. As reported in Table 3, the RiLK1-analog peptide RiLK3 showed a bactericidal activity equal to that of RiLK1 against *E. coli* (MBC = 2.0 μM) and *P. aeruginosa* (MBC = 50 μM) strains. In contrast, RiLK3 showed a lesser bactericidal activity than its parental RiLK1 against *L. monocytogenes*, *S. typhimurium* and *S. aureus*, with MBC values 9-, 2-and 4.5-times higher than those of RiLK1, respectively.

**Table 3:** Antimicrobial properties of the mutated peptide RiLK3 in comparison with those of the parent RiLK1.

Strain	RiLK1	RiLK3
	MBC (μM)	MBC (μM)
*P. aeruginosa* (ATCC 27853)	50.0	50.0
*S. typhimurium* (wild strain)	2.5^a^	5.0
*E. coli* (ATCC 25922)	2.0^a^	2.0
*S. aureus* (ATCC 25923)	16.0^a^	75.0
*L. monocytogenes* (wild strain)	2.0^a^	18.0

a Agrillo et al. (2020).

**Figure 1:**
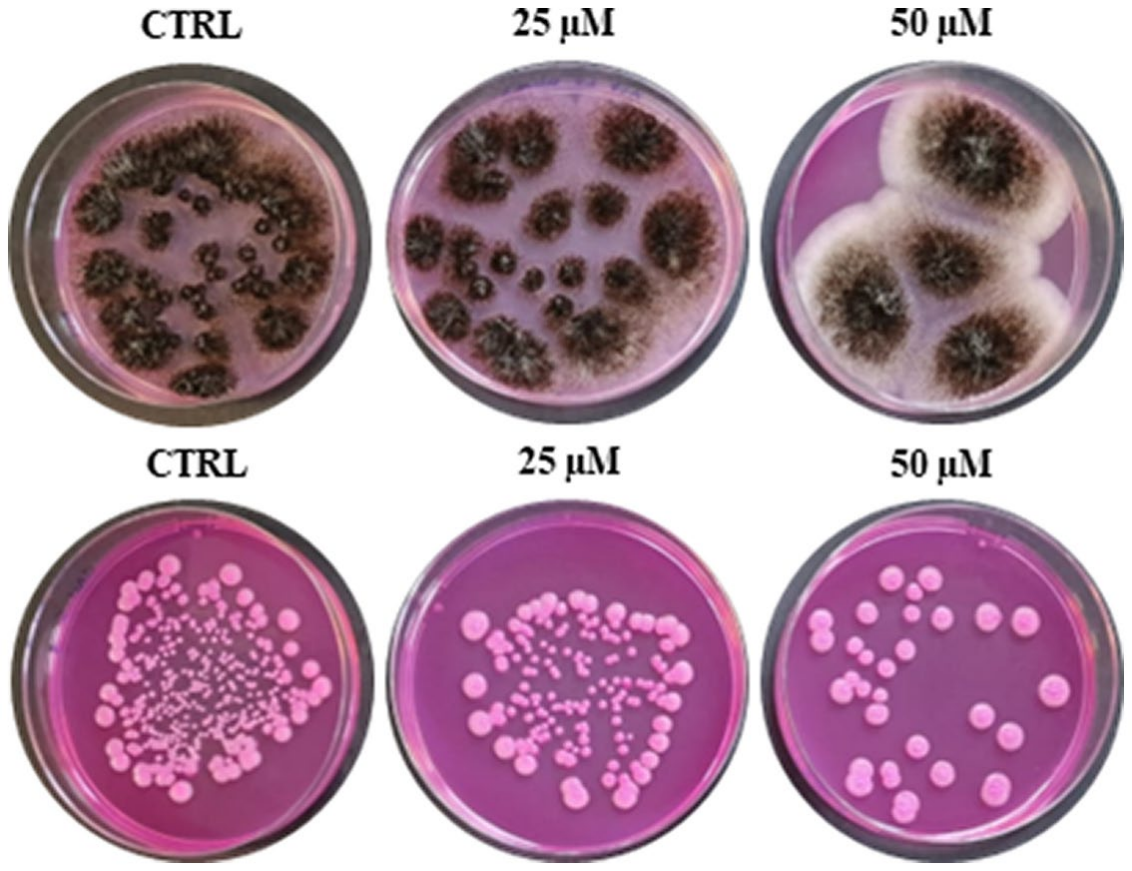
Antifungal activity of RiLK3 against two pathogenic fungi. **(A)** *Aspergillus brasiliensis* and **(B)** *Candida albicans*. CTRL: each tested pathogen without peptide treatment. The fungal cultures (1×10^5^ CFU/mL) treated or not with different peptide concentrations (25 and 50 μM) for 6 h at 37°C, were seeded on DG18 plates. The photographs are representative of three independent experiments performed in triplicate.

Due to the increasing incidence of drug-resistant fungi and the limitations of existing treatment strategies for infections caused by fungi, the fungicidal activity of RiLK3 (Figure 1) was also evaluated *in vitro* against two of the most common fungal pathogens such as *A. brasiliensis* and *C. albicans* and compared with that previously determined for RiLK1 (Agrillo et al., 2020). The antifungal susceptibility testing clearly revealed that RiLK3 was less active than its parent, inhibiting only ~87% growth of *A. brasiliensis* and ~90% growth of *C. albicans* even at the highest concentration tested (50 μM), whereas RiLK1 at 25 μM concentration had a total inhibitory effect on the growth of both fungi (MFC) (Agrillo et al., 2020).

Finally, the antiviral effects of RiLK1 and RiLK3 were investigated *in vitro* for the first time against the hepatitis A virus (HAV) to assess whether the lysine to arginine substitution affected the virucidal activity. Indeed, HAV is a non-enveloped single-stranded RNA virus provoking acute hepatitis in humans, a worldwide infectious disease. For this reason, the development of new antivirals against HAV may be important for the control of viral infections. Firstly, the cytotoxicity of the two peptides on the Frp3 cell lines was assessed at different concentrations, revealing that both molecules did not have any effect on the viability of Frp3 cell line at all the doses tested (data not shown). Therefore, the antiviral assays were performed and the results of the virucidal effects are summarised in Table 4. Interestingly, HAV treated with RiLK1 showed a decrease in viral infectivity greater than 1 log in comparison with the untreated virus at both 80 μM (1.4 log) and 40 μM (1.1 log) concentrations, corresponding to a reduction in the infectious potency of 96.6% and 93.3%, respectively. Conversely, RiLK3 was unable to inhibit HAV infection at the same concentrations tested.

**Table 4:** *In vitro* effect of RiLK1 and RiLK3 peptides on HAV infectivity by calculating log reduction value (LRV).

	Viral titre after treatment	Log reduction value
	(logTCID50/mL ± SD)	(logTCID50/mL ± SD)
Untreated HAV	4.7 ± 0.2	
RiLK1 (80 μM)	3.3 ± 0.2	1.4 ± 0.4
RiLK1 (40 μM)	3.6 ± 0.1	1.1 ± 0.3
RiLK3 (80 μM)	4.4 ± 0.1	0.3 ± 0.3
RiLK3 (40 μM)	4.4 ± 0.1	0.3 ± 0.3

Therefore, these findings are interesting in light of other works in which it is reported that increased arginine content not necessarily determine an improvement of the AMP activity, providing further indications for the design of new antimicrobial peptides (Hristova and Wimley, 2011; Cutrona et al., 2015).

### 3.3. Structural characterization of RiLK3

Studies of peptide-detergent interaction are very important for AMP research taking into consideration the peculiarity of their mechanism of action activity, which is usually via bacterial membrane disturbance (Hancock and Rozek, 2002; Benfield and Henriques, 2020). Commonly, this interaction induces conformational changes to the peptides themselves, which are mainly unstructured in solution. In this context, CD spectroscopy was performed to understand the role of secondary structural features on the antimicrobial potency of RiLK3 peptide in comparison with its parental RiLK1, using the negatively charged SDS as a prokaryotic membrane-mimetic model.

**Figure 2:**
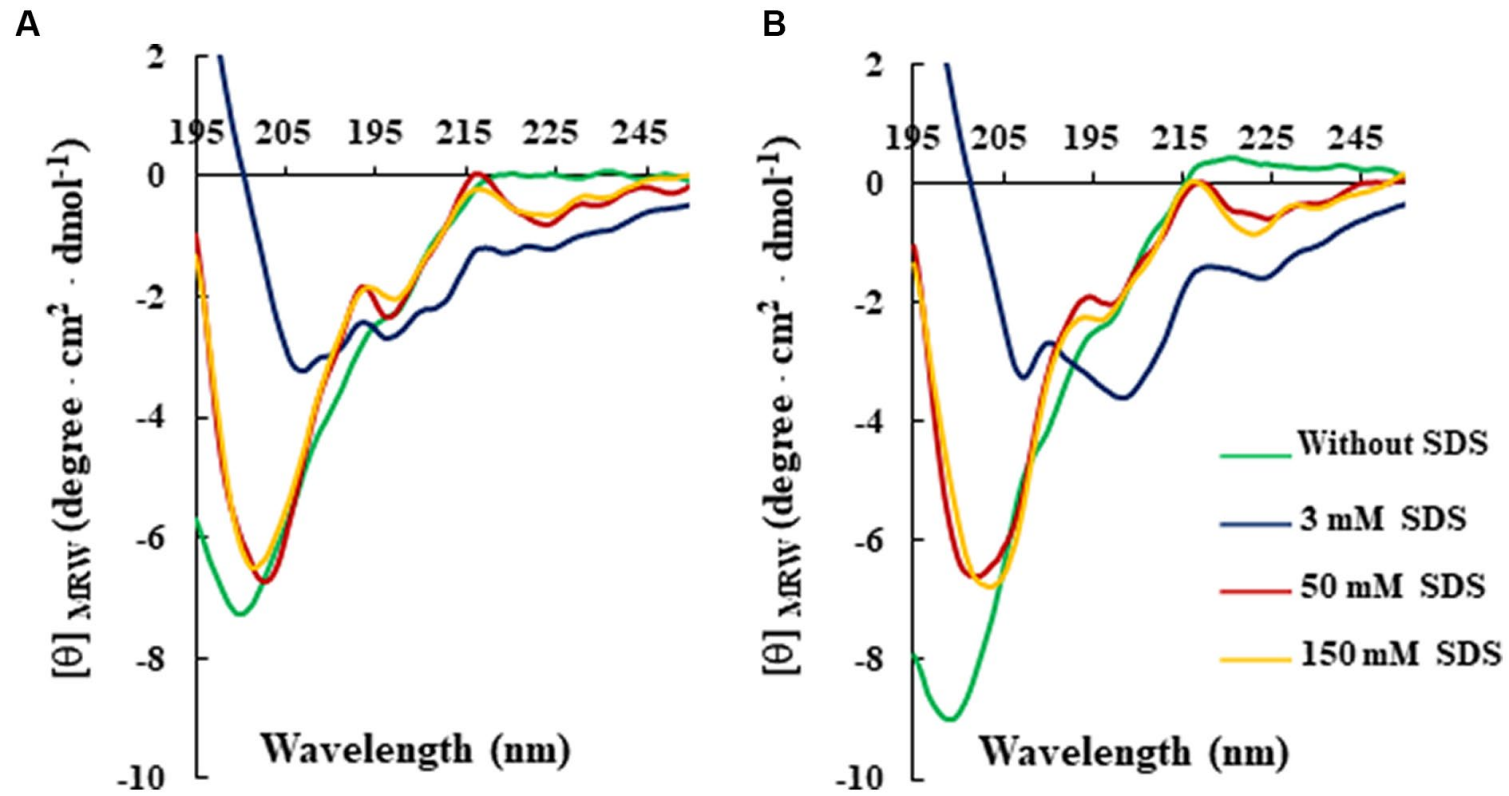
Effect of SDS concentration on the secondary structure of RiLK3 and RiLK1 monitored by circular dichroism. Far-UV CD spectra of **(A)** RiLK3 and **(B)** RiLK1. All spectra were recorded at a peptide concentration of 80 μM in 10 mM Tris-HCl. pH 7.0 and at 25°C in the absence (green lines) or presence of SDS at different concentrations.

**Figure 3:**
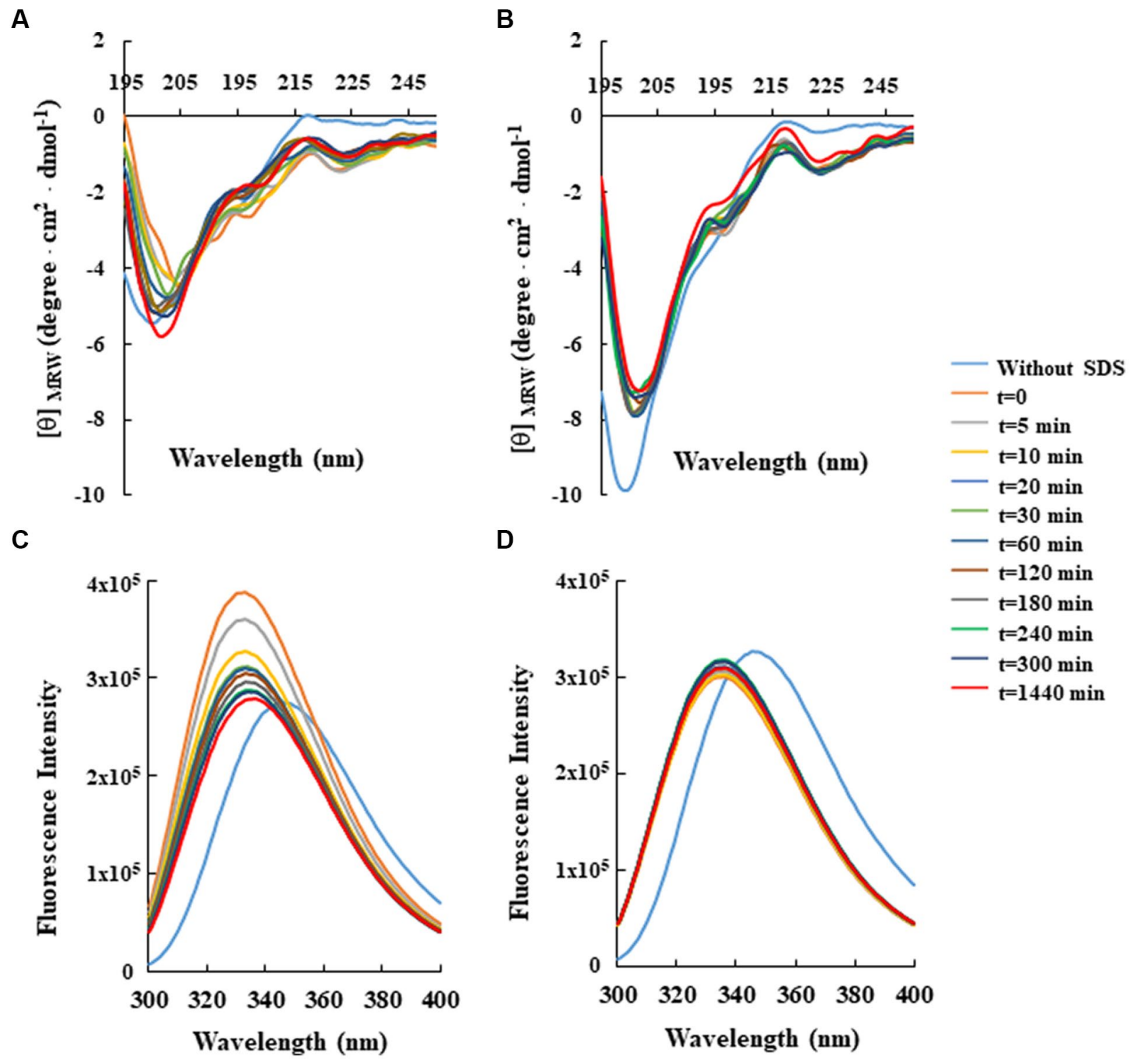
Time-dependent effect of SDS on the secondary and tertiary structure of RiLK3 and RiLK1 monitored by spectroscopic techniques. Far-UV circular dichroism spectra of **(A)** RiLK3 and **(B)** RiLK1. Intrinsic fluorescence emission spectra of **(C)** RiLK3 and **(D)** RiLK1. All spectra were recorded at a peptide concentration of 80 μM in 10 mM Tris-HCl. pH 7.0 in the presence or absence (blue lines) of SDS (50 mM) during 24 h incubation at 25°C.

For this analysis, CD spectra of both peptides were recorded at a constant concentration (80 μM) in 10 mM Tris-HCl buffer (pH 7.0) and in the absence or presence of SDS solutions, below and above the critical micelle concentration (cmc). As shown in Figure 2, the CD spectra of RiLK3 (Figure 2A) and RiLK1 (Figure 2B) showed a pronounced negative band below 200 nm in a water solution, indicating a predominantly random coil secondary structure, typical of an unstructured peptide. After adding increasing concentrations of SDS (3–150 mM), both peptides exhibited CD spectra very similar in shape when in contact with the oppositely charged amphiphile. Specifically, in solutions at the SDS concentration below cmc (3 mM), RiLK3 and RiLK1 adopted mainly α/β-mixed conformations, which underwent a dramatic shape change when the detergent was present in micellar concentration (50 and 150 mM). As depicted in Figure 2, both peptides showed a more complex folded conformation, that was not correlated with the common secondary structure elements (α-helix, β-strand, or random coil), and that could be due to the co-existence of multiple α/β-like subpopulations and/or the propensity to form higher-ordered self-aggregates, which assemble into bigger oligomeric species in equilibrium with partially structured monomers. Subsequently, the folding kinetics of RiLK3 and RiLK1 were monitored in the presence of 50 mM SDS during 24 h incubation. The CD spectra (Figures 3A,B) evidenced that each peptide retained its own complex conformational distribution during the time, as confirmed by the assessment of the secondary structure elements performed by the K2D software (Supplementary Table S1). Therefore, from the structural point of view, the modification of the identity of the basic residues did not induce appreciable changes in the secondary structure of the mutant in comparison to the wild-type peptide.

To corroborate the CD data, the same analyses were carried out by fluorescence, taking advantage of the occurrence of two tryptophan residues in both sequences. In aqueous solution, the maximal fluorescence emission (*λ*_max_) for the two peptides was observed at ~350 nm, a value that is typical for the Trp indole group fully exposed to hydrophilic environments and that is consistent with the disordered secondary structure observed at this condition by CD (Figures 3C,D). Immediately after SDS addition at 50 mM concentration (*t* = 0), an increase in the quantum yield of fluorescence accompanied by a concomitant blue shift of *λ*_max_ from 350 to 335 nm, was observed for RiLK3, indicative of a reduction in the polarity around the Trp residues and an ordered structural reorganisation. As the incubation time increased, the fluorescence intensity gradually decreased, suggesting a strong involvement of one or both Trp residues in hydrophobic interactions with the detergent micelles, resulting in their shielding (Figure 3C). Another source of quenching might be the interaction between tryptophan and the tryptophan-flanking lysine in the peptide under investigation (Zhao and Kinnunen, 2002), like. Moreover, the tryptophan residues could suffer from fluorescence self-quenching if peptide oligomerization takes place upon binding, as also evidenced by CD spectra.

Concerning RiLK1, the addition of SDS shifted *λ*_max_ to lower wavelengths without affecting the fluorescence intensity, which remained constant for the whole incubation period (24 h). This behaviour could be due to a fast saturation of Trp fluorescence, indicative of reaching a stationary phase in which the peptide does not remain really “stationary” but it continuously modifies its highly dynamic supramolecular conformations with time.

Next, the effect of pH and temperature-effects on peptide-SDS complexes were investigated, being both physicochemical parameters that strongly influence the AMP efficacy. As shown in Supplementary Figures S1, S2, the changes in pH or temperature monitored up to 48 h did not markedly affect the structural and folding stability of RiLK3 when in complex with SDS micellar solutions in the experimental range analysed. The same behaviour was also observed with RiLK1 (Supplementary Figures S3, S4), confirming the ability of both molecules to adapt to different environmental conditions and therefore to retain their antimicrobial activity at different temperatures and pH values. Remarkably, a structural-functional correlation does not emerge in this study as the weakened activity of the arginine mutant is not accompanied by distinctly different structures than those observed for the lysine mutant, as evidenced by our spectroscopic data.

### 3.4. Oligomerization of RiLK3 or RiLK1 in SDS micelles

**Figure 4:**
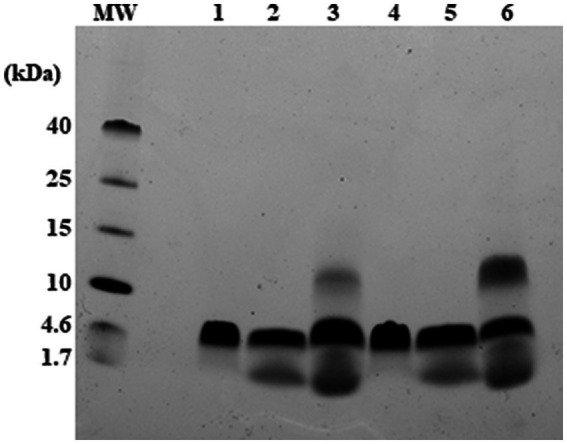
Analysis of RiLK3 and RiLK1 oligomerization in SDS micelles by electrophoresis. Tris-tricine SDS-PAGE (17%) of RiLK1 or RiLK3 at 240 μM concentration treated or not with glutaraldehyde (4% concentration) in presence of SDS (150 mM) for 24 h at 37°C. MW: molecular weight references; lane 1: RiLK1; lane 2: RiLK1 + SDS (150 mM); lane 3: RiLK1 + SDS + glutaraldehyde; lane 4: RiLK3; lane 5: RiLK3 + SDS; lane 6: RiLK3 + SDS + glutaraldehyde. A 10 μL amount of each sample was loaded onto the gel.

The tendency of RiLK1 and RiLK3 to oligomerize in the presence of SDS micelles was also assessed by performing glutaraldehyde-mediated cross-linking experiments. At high concentrations of glutaraldehyde, both peptides demonstrated to form predominantly trimeric states as evidenced by the presence of a prominent SDS-PAGE band corresponding to molecular mass of ~5 kDa upon incubation of peptide/SDS with the cross-linker (Figure 4). Moreover, cross-linked species belonging to larger (about 12 kDa) or lesser (about 1.7 kDa) molecular masses corresponding to monomeric state, were also seen, albeit with minor populations. However, upon treatment with SDS micelles in the absence of glutaraldehyde a detectable oligomerization corresponding to the trimeric state of RiLK3 and RiLK1 was observed. This behaviour could indicate a covalent association among the peptide molecules as it was not disrupted by SDS detergent unlike the high-mass oligomers, which may be non-covalent nature. It is worth noting that both peptides, RiLK3 and RiLK1, appeared to migrate on the SDS-PAGE as trimeric species in the absence of cross-linker and SDS micelles, thus suggesting a propensity of peptides to self-assembled.

**Figure 5:**
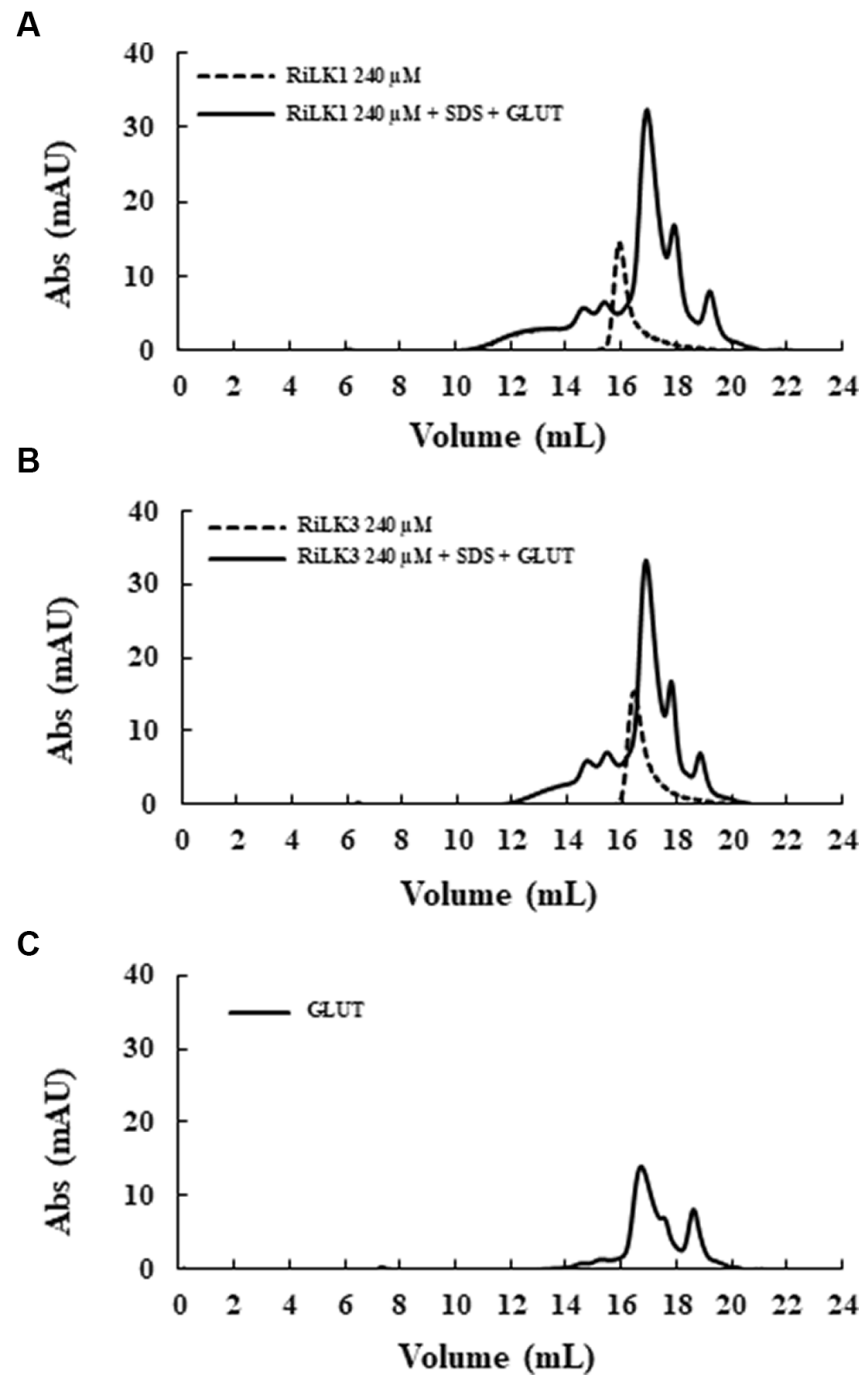
Gel filtration chromatography of the crosslinked products of RiLK3 and RiLK1 in presence of SDS micelles. Size exclusion chromatography was performed on the Superdex 30 Increase column, pre-equilibrated with 50 mM Tris-HCl buffer (pH 7.5) containing 150 mM NaCl and 20% acetonitrile. **(A)** RiLK1 (240 μM) incubated at 37°C for 24 h with SDS (150 mM) in presence of glutaraldehyde (4%). **(B)** RiLK3 (240 μM) incubated at 37°C for 24 h with SDS (150 mM) in presence of glutaraldehyde (4%). **(C)** Glutaraldehyde (4%) incubated at 37°C for 24 h. After incubation, all the samples were analysed by gel filtration. The dashed lines represent the peptide solutions (240 μM) incubated at 37°C for 24 h without SDS and glutaraldehyde. The reported chromatograms are representative of three independent experiments.

To gain insights into the oligomerization propensity of the two peptides in the presence of membrane mimics, size exclusion chromatography was performed on the same samples analysed by SDS-PAGE. As depicted in Figure 5, in the absence of detergent and glutaraldehyde, monomers of RiLK1 and RiLK3 were observed. After adding the cross-linker, a homogeneous population containing peptide species with high molecular weights was present in the cross-linked samples, as demonstrated also by the fluorescence assays performed on the fractions eluted and collected from the size-exclusion column (data not shown). The obtained results confirmed the oligomerization behaviour of both AMPs when in contact with membrane-like environments.

### 3.5. FT-IR analysis of RiLK1 and RiLK3

**Figure 6:**
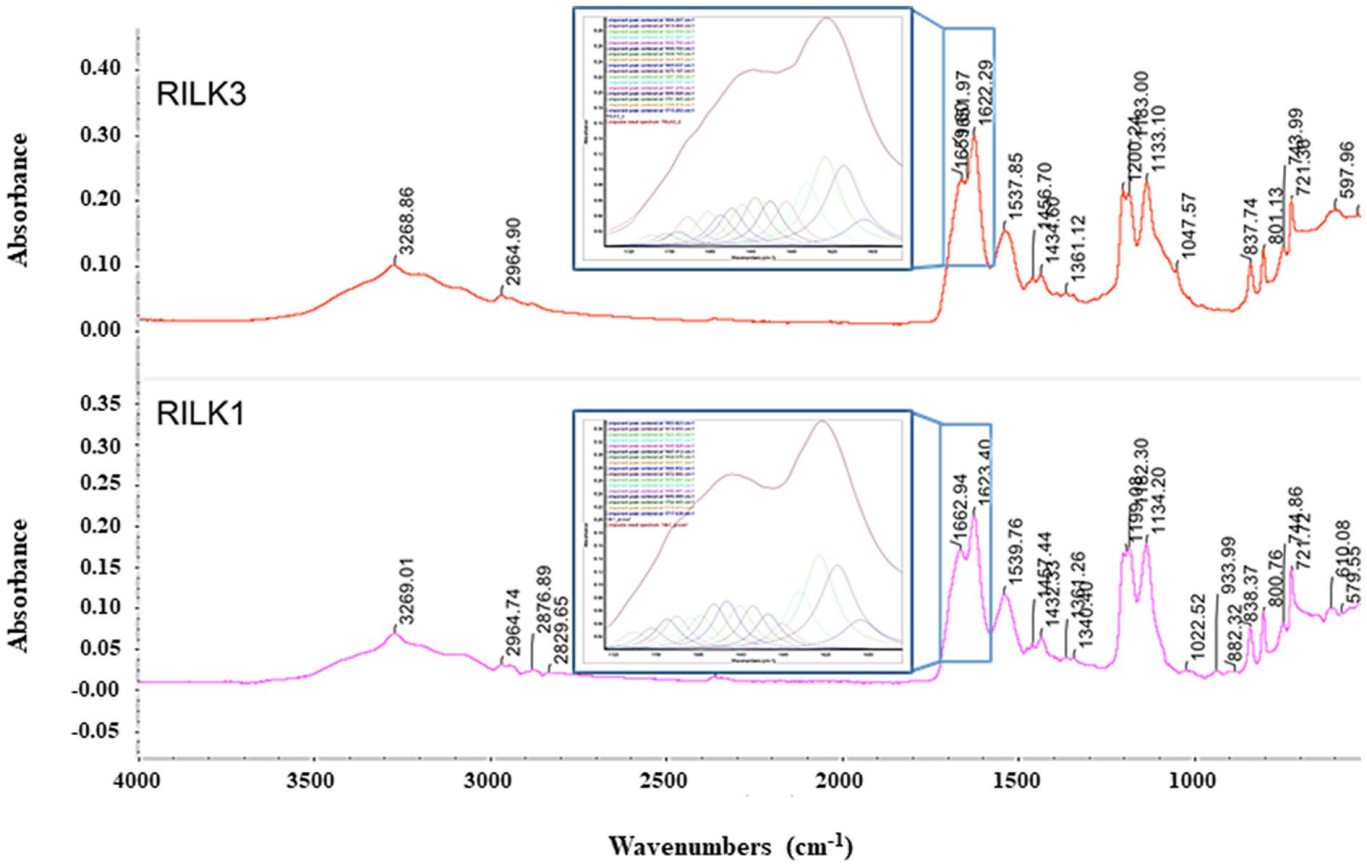
FTIR absorption spectra of RILK1 and RILK3 in powder form. Main peaks are underlined. Into the boxes, the amide I bands of RILK1 and RILK3, respectively with their deconvolution data analysis, were reported.

To obtain further information on the possible self-assembly propensity of RiLK1 and RiLK3, the Fourier transform infrared spectroscopy (FT-IR) was applied in the range from 4000 to 525 cm^−1^ (Wi et al., 1998). Usually, polypeptides show nine characteristic IR absorption bands: amide A and B around 3300 and 3100 cm^−1^, respectively, attributed to NH stretching; amide I in the range of 1600–1700 cm^−1^ responsible for C=O stretching; amide II and III in the range 1480–1575 cm^−1^ and 1229–1301 cm^−1^, respectively, attributed to CN stretching and NH bending; amide IV in the range 625–767 cm^−1^, attributed to OCN bending; amide V in the range 640–800 cm^−1^, attributed to out of plane NH bending; amide VI in the range 537–606 cm^−1^, attributed to out of plane C=O bending; amide VII around 200 cm^−1^, attributed to skeletal torsion (Wi et al., 1998; Rea et al., 2014; Portaccio et al., 2015). Specifically, the universally available amide I band (1600–1700 cm^−1^) is the most utilised probe for the estimation of the secondary structural composition and conformational changes of peptides, due to the high sensitivity of the C=O stretching frequency to small changes in molecular geometry and hydrogen bonding pattern, i.e., to each secondary structure (Rea et al., 2014; Portaccio et al., 2015). In this context, RILK1 and RILK3 present the typical peptide spectrum with almost all amide bands previously described. Specifically, the absorption spectra of RILK1 and RILK3 analysed in powder form are reported in Figure 6 while the assignment of the peaks is presented in Supplementary Table S2. Moreover, a more detailed analysis on the structure was provided by the second derivative and decomposition of the amide I band into sub-bands. The deconvolved spectra were fitted with Lorentzian bands, whose results are resumed into the boxes of Figure 6 and in Supplementary Tables S3, S4. Any peaks correspond to a C=O stretching frequency that can be linked to a specific secondary structure. As shown in Figure 6, the absorption spectrum of RILK1 in powder form presents a large double peak in the range 1600–1700 cm^−1^, with a relative maximum at 1622 cm^−1^, usually attributed to β-sheet, and at 1663 cm^−1^, usually attribute to 310-Helix. The deconvolved spectra allowed us to carry out a quantitative analysis of the content of the secondary elements present in the RILK1 structure. Specifically, it was found that structures of type β are responsible for 74.3% of the vibrational modes into the peptide (Supplementary Table S3). Similarly, also absorption spectrum of RILK3 in powder form presents a large peak in the range 1600–1700 cm^−1^, with relative maxima at 1622 cm^−1^, usually attributed to β-sheet, at 1652 cm^−1^, usually attribute to α Helix, and at 1660 cm^−1^, usually attribute to 310-Helix. The deconvolved spectra revealed that structures of type β are responsible for 69.3% of the vibrational modes into RILK3, while α type components are only 11.9% (Supplementary Table S4).

### 3.6. Binding assays

**Figure 7:**
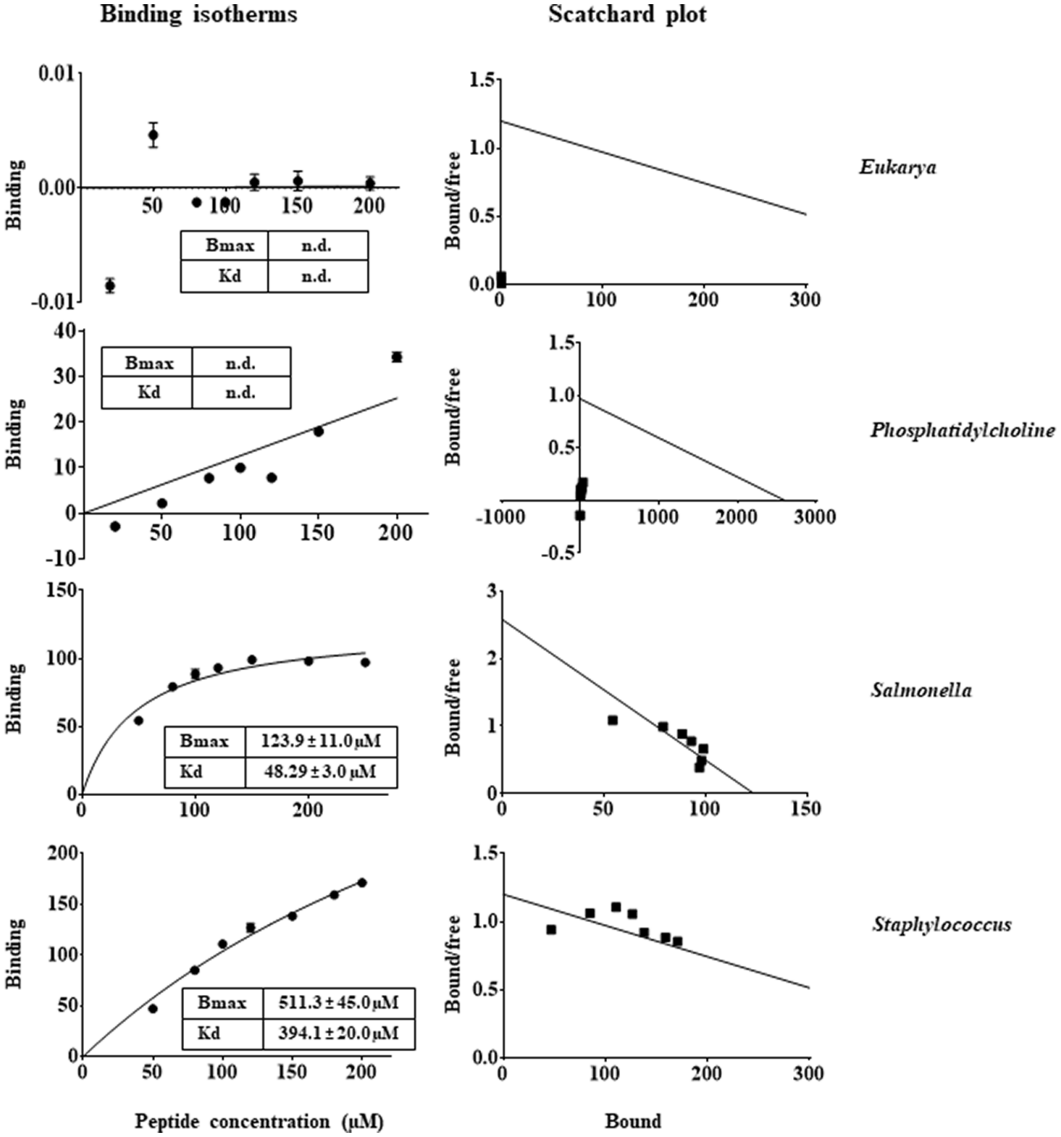
Trp fluorescence analysis of binding of RiLK1 to MLVs. Binding isotherms calculated from Trp fluorescence intensity at 335 nm of RiLK1 (1 μM) with model membrane vesicles (30 μM) in HEPES buffer with NaCl 150 mM. Scatchard plot analysis for the binding data of RiLK1. Data are presented as means ± s.d. of different samples analysed in quadruplicate. n.d. the equation could not fit the data, not possible to determine.

**Figure 8:**
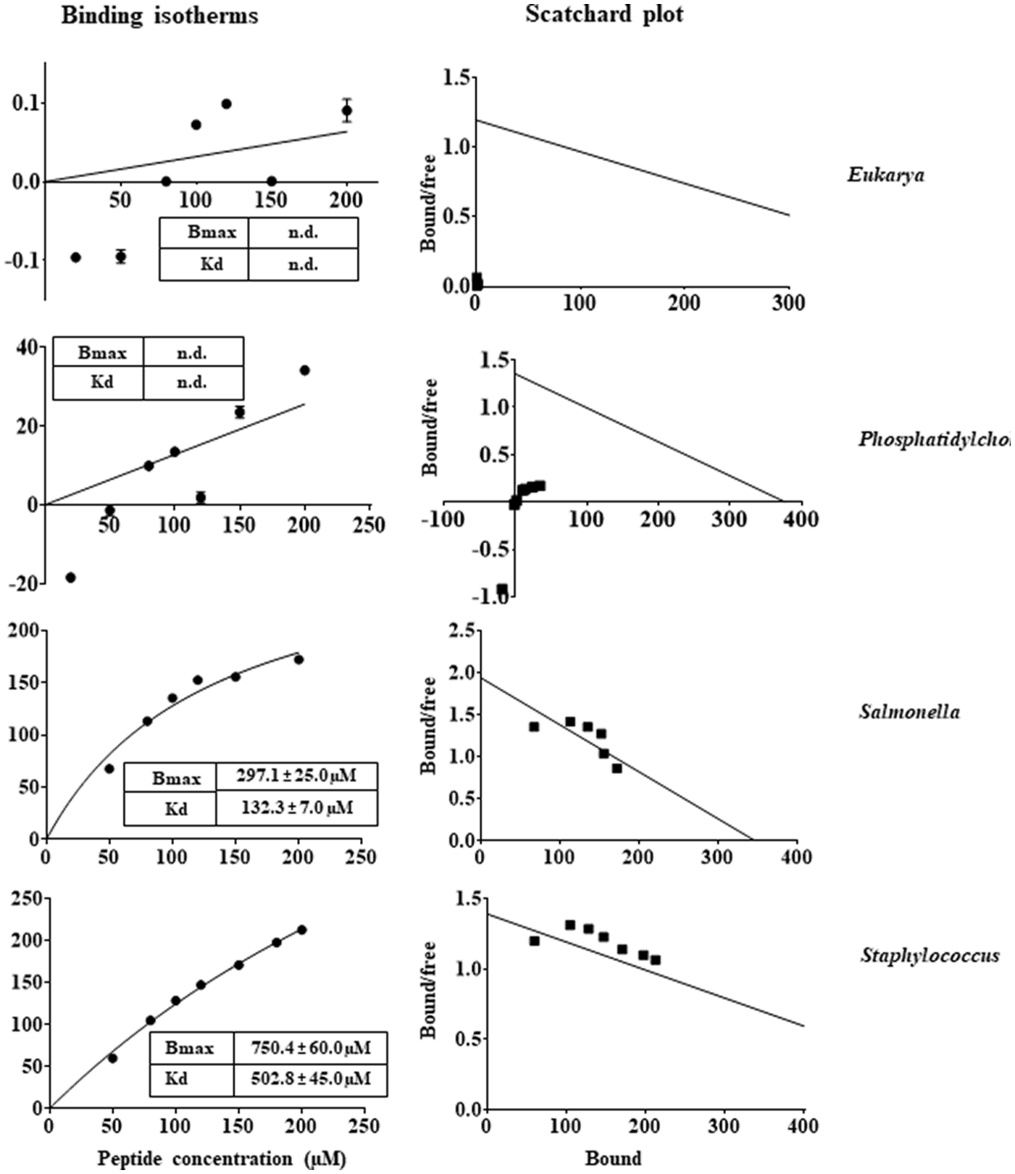
Trp fluorescence analysis of binding of RiLK3 to MLVs. Binding isotherms calculated from Trp fluorescence intensity at 335 nm of RiLK3 (1 μM) with model membrane vesicles (30 μM) in HEPES buffer with NaCl 150 mM. Scatchard plot analysis for the binding data of RiLK3. Data are presented as means ± s.d of different samples analysed in quadruplicate. n.d. the equation could not fit the data, not possible to determine.

In general, the primary target of most natural AMPs (Huang et al., 2004; Lohner and Blondelle, 2005) is recognised to be the bacterial membranes that typically contain negatively charged phospholipids, which promote the binding of the cationic peptides (Zasloff, 2002; Jenssen et al., 2006; Lohner et al., 2008; Lohner, 2009). This could be the reason of the highly selectivity of AMPs towards microorganisms, in view of the differences between the lipid composition of mammalian and bacterial membranes. Therefore, membrane interaction emerged as a key factor to consider the mode of action of these peptides (Matos et al., 2010). In this context, in order to investigate the effects that RiLK1 and RiLK3 exerted at the level of the cell membrane, a study of the interaction between the two peptides and biomimetic model systems of variable lipid compositions was performed by monitoring changes in the intrinsic fluorescence of Trp. These studies also provide valuable information about the molecular basis of the membrane lipid-peptide interaction, as the electrostatic binding may cause shallow surface peptide depots (plaque formation) or intercalation into (pore formation) the lipid bilayer. Upon binding to negatively charged MLVs resembling the membranes of the Gram-negative *Salmonella* and the Gram-positive *Staphylococcus*, a blue shift of the maximum wavelength in the peptide Trp emission spectra was observed in both the lipid environments, indicating the transfer of Trp residues in peptides from the aqueous phase to a more hydrophobic environment in lipid membranes. On the contrary, there was small or no blue shift when the peptides were added to zwitterionic or neutral MLVs, indicative of a negligible partitioning of the peptides into these membranes. From the binding isotherms of RiLK1 (Figure 7) and RiLK3 (Figure 8) upon binding to negatively charged lipid vesicles as a function of lipid-to-peptide molar ratio and the linear regression to the one-site binding model (Figures 7, 8), the dissociation constant *K*_d_ and the maximal binding capacity *B*_max_ were obtained. It is worth noting that RiLK1 displayed a much higher affinity to the bacterial membranes than RiLK3, thus suggesting differences in the membrane mode interaction of the two peptides, although RiLK1 bound about 8-fold more tightly to anionic *Salmonella* than to anionic *Staphylococcus* bilayers, probably due to their distinct lipid compositions and to the surface charge of the membranes. Therefore, this result shows a higher affinity in the binding of RiLK1 to Gram-negative bacterial membranes compared to Gram-positive lipid bilayers. This would be consistent with the hypothesis that the Gram-negative bacteria are more easily killed by RiLK1 because their membranes have a high concentration of zwitterionic lipids together with anionic lipids. This result is supported by *in vitro* antimicrobial assays previously described, where the Gram-negative bacterial species *S. typhimurium* that has a high content of the zwitterionic lipid DOPE, also has MBC value that is substantially lower than that determined for the Gram-positive *S. aureus,* whose membrane lipids are largely anionic (POPG and CL) and are devoid of uncharged lipids.

In line with the *K*_d_ values of RiLK1 binding to these membranes, *B*_max_ values were smaller than those of RiLK3, demonstrating the higher affinity of RiLK1 with respect to RiLK3 for bacterial membranes. However, saturation upon binding of both peptides to the *Staphylococcus-*like membranes was not achieved under the experimental conditions used, thus indicating that a higher ligand concentration was required and suggesting that RiLK1 and RiLK3 bound less specifically to this type of membrane. Conversely, no changes in the binding curves were observed in the presence of MLVs of PC or PC:POPE 40:40 mimicking zwitterionic and eukaryal membranes (Figures 7, 8), demonstrating that RiLK1 and RiLK3 had essentially no affinity for these uncharged membranes and suggesting that the electrostatic forces play a crucial role in the membrane-peptide interaction, thus driving the preference of the two AMPs for the bacterial cells over eukaryotic ones.

In conclusion, it is clear that increasing the arginine content as in RiLK3 (5 arginine residues compared to 4 in RiLK1) did not enhance its interaction with prokaryotic membranes and its antimicrobial activity. Therefore, it is likely that an optimal number of arginine residues at the specific positions along the peptide sequence can represent the driving force for an efficient cell-penetrating ability of arginine-rich peptides.

### 3.7. Dynamic light scattering and ζ-potential analysis

**Figure 9:**
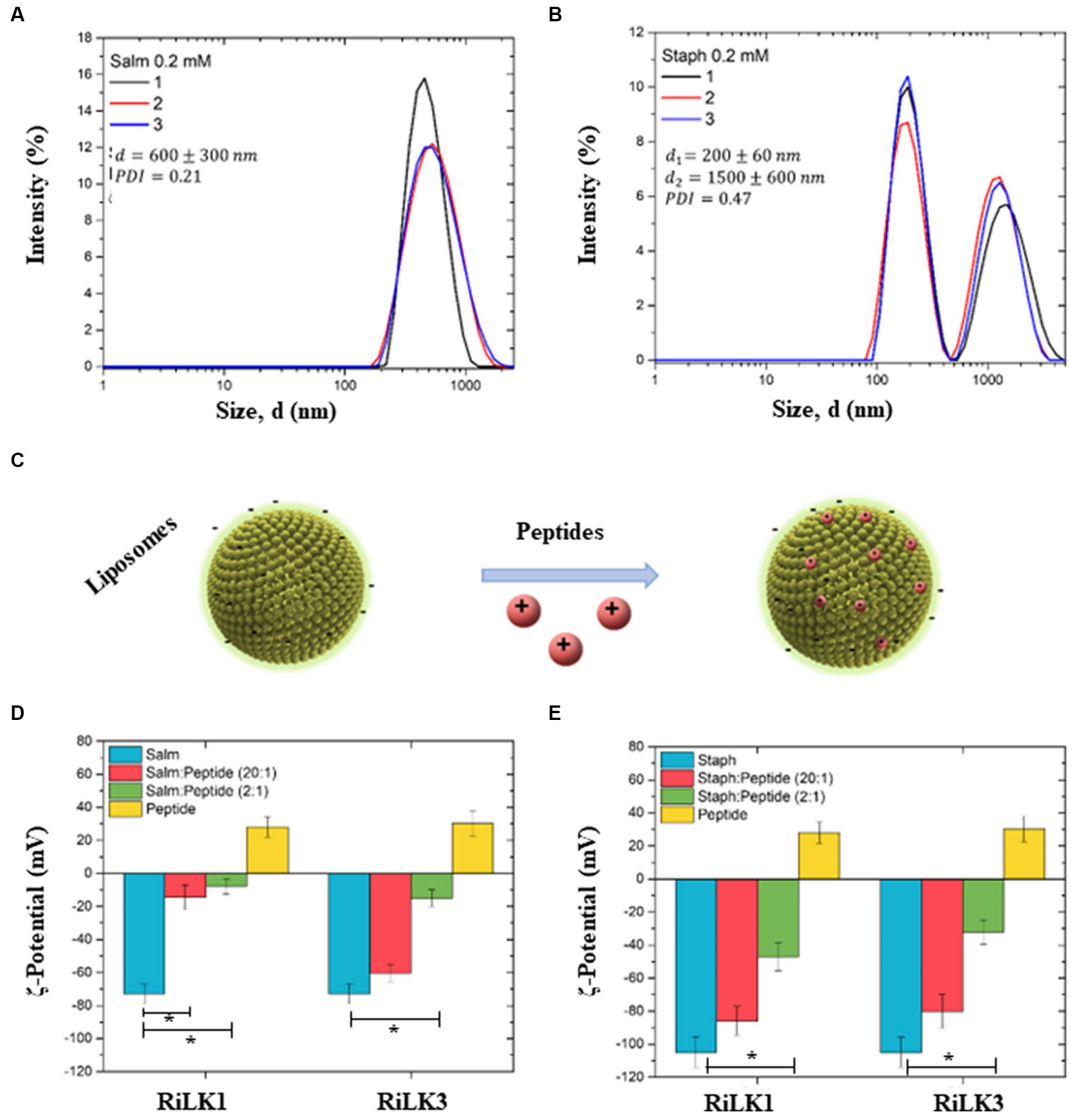
Dynamic light scattering and ζ-potential analysis of bacterial membrane liposomes. **(A)** Hydrodynamic size distribution (*d*) of *Salmonella*-like liposomes (0.2 mM). The mean d value, and the PDI are reported (*n* = 3). **(B)** Hydrodynamic size distribution of *Staphylococcus*-like liposomes (0.2 mM). Two main populations, whose mean sizes are denoted as *d*_1_ and *d*_2_, are identified as wells as the PDI is reported (*n* = 3). **(C)** Schematic representation of the interaction between liposomes (*Salmonella* and *Staphylococcus*) and peptides (RiLK1 and RiLK3). **(D)** ζ-potential histograms of *Salmonella* liposomes (blue), interacting with RiLK1 and RiLK3 peptides in liposome:peptide ratio of 20:1 (red) and 2:1 (green). ζ-potential of the peptides alone are also reported (yellow). **(E)** ζ-potential histograms of *Staphylococcus* liposomes (blue), interacting with RiLK1 and RiLK3 peptides in liposome:peptide ratio of 20:1 (red) and 2:1 (green). ζ-potential of the peptides alone are also reported (yellow). ^*^Significant differences (*p* < 0.05) between the bacterial-like liposomes alone (light blue) and the bacterial-like liposomes in the presence of the peptides (red or green).

To further assess the effects of RiLK1 and RiLK3 on the overall structure of bacterial membranes, a preliminary physical-chemical characterization of our liposomal model systems, with or without AMPs, was carried out by performing dynamic light scattering (DLS) and ζ-potential measurements. DLS measurements of the *Salmonella*-like liposome at a concentration of 0.2 mM showed a monodisperse distribution with mean size (*d*) of 600 ± 300 nm and a polydispersity index (PDI) of 0.21 (Figure 9A). In the same way, DLS measurements were performed on *Staphylococcus*-like liposomes, revealing the presence of two main populations: the first population exhibited a mean size (*d*_1_) of 200 ± 60 nm, while the second one exhibited a mean size (*d*_2_) of 1500 ± 600 nm (Figure 9B). Due to the presence of the two populations, the measured PDI value increased to 0.47, as compared to the one obtained for *Salmonella*-like liposomes. Then, ζ-potential measurements were carried out to highlight the interactions between the peptides (RiLK1 and RiLK3) with both bacterial-mimic liposome types (Figure 9C). First, the ζ-potential of *Salmonella-*mimic liposome and both peptides, before and after the interaction in two different ratios (liposome:peptide 20:1 and 2:1, respectively), was measured. *Salmonella*-like liposomes exhibited a highly negative surface charge (−73 ± 6 mV), guaranteeing sufficiently high electrostatic repulsion among the liposomes and avoiding the formation of clusters and/or precipitates (Figure 9D—blue bars). Therefore, the suspension resulted to be highly stable. RiLK1 and RiLK3 peptides exhibited, instead, a positive surface charge (28 ± 6 mV and 30 ± 8 mV, respectively) and resulted to be stable in solution (Figure 9D—yellow bars). The interaction of *Salmonella*-like liposomes with RiLK1 and RiLK3 peptides in a ratio of 20:1 (Figure 9D—red bars) and 2:1 (Figure 9D—green bars) was assessed by the net surface charge variation. More precisely, the interaction of *Salmonella*-mimic liposomes with RiLK1 caused a high change in the net charge measurement for both interaction ratios. Differently, RiLK3 needed a ratio of 2:1 to significantly affect the surface charge of *Salmonella*-like liposomes. This corresponds to a higher peptide concentration needed to be effectively available on the liposome surface.

Analogously, the ζ-potential of *Staphylococcus*-mimic liposomes standing alone and after the interaction with the two peptides in the same ratios chosen for *Salmonella*-mimic liposomes (20:1 and 2:1, respectively), was measured. *Staphylococcus*-like liposomes exhibited a higher negative surface charge (−105 ± 9 mV) than those of *Salmonella*, (Figure 9E—blue bars). Therefore, the suspension resulted to be highly stable. RiLK1 and RiLK3 positive surface charges are reported in Figure 9E (yellow bars) for comparison. Also, in this case, the interaction of *Staphylococcus*-like liposomes with RiLK1 and RiLK3 peptides in a ratio of 20:1 (Figure 9E—red bars) and 2:1 (Figure 9E—green bars) was assessed by the net surface charge variation. Differently from the results obtained from the interaction of *Salmonella*-mimic liposomes with RiLK1 or RiLK3, a lower change in the net charge measurements was observed for both peptides at a liposome:peptide ratio of 20:1 (Figure 9E—red bars). A higher variation was observed for both peptides at higher peptide concentrations (ratio 2:1), as reported in Figure 9E (green bars).

## 4. Conclusion

In this study, the already characterised decapeptide RiLK1 was modified through the substitution of the positively charged residue Lysine into Arginine, obtaining the mutant peptide RiLK3. The antimicrobial analysis demonstrated that the derivative RiLK3 displayed lower or in some cases negligible antibacterial, antifungal, or antiviral activity compared to its parent, despite they did not exhibit any evident differences from the structural point of view. Moreover, as the first proof of evidence, experimental analyses with model lipid vesicles highlighted that increasing the composition of arginine versus lysine residue did not improve membrane interaction of RiLK3 mutant towards bacterial-like anionic membranes.

This work demonstrates that *de novo* generation of AMPs is still not a trivial endeavour and the prediction of peptide characteristics needs to be made on a case-by-case. Moreover, the results evidence that just one peptide (RiLK1) is able to be active against three different types of pathogens, thus suggesting diverse modes of action, as reported also for the human cathelicidin LL-37 (Barlow et al., 2011; Wong et al., 2011). Indeed, while antibacterial activity could be deduced by the liposome binding assays, the action against viruses and fungi is much less comprehensible. It is likely that RiLK1 is targeted against highly preserved structures, e.g., the phospholipid membrane or other constitutive components like peptidoglycans in Gram-negative and Gram-positive bacteria, or glucan in the fungal cell wall. Concerning the antiviral activity, some preliminary indications lead us to speculate that the peptide may act directly on the hepatitis virion rather than on the host cell. However, further investigations are in progress to better elucidate the mechanisms of action of RiLK1 as antiviral and antifungal agent.

## Data availability statement

The raw data supporting the conclusions of this article will be made available by the authors, without undue reservation.

## Author contributions

AP, LG, TV, AM, BM, and BA performed the experiments. YP, GP, and LC: funding acquisition. GP, MG, PD, and LC: data curation, formal analysis, and writing original draft. PE and MB: software, designed the tables and figures and review. All authors contributed to the article and approved the submitted version.
