# Supplementary material for: Antimicrobial activity, membrane interaction and structural features of short arginine-rich antimicrobial peptides
Source: Front Microbiol. 2023 Oct 5;14:1244325. doi: 10.3389/fmicb.2023.1244325 (PMC10585156; doi:10.3389/fmicb.2023.1244325)
Supplement: Supplementary file 1 [file Data_Sheet_1.docx]

***Supplementary Material***

**Antimicrobial activity, membrane interaction and structural features of short arginine-rich antimicrobial peptides**

Bruna Agrillo^1,†^, Alessandra Porritiello^2,†^, Lorena Gratino^2^, Marco Balestrieri^2^, Yolande Therese Proroga^3^, Andrea Mancusi^3^, Loredana Cozzi^4^, Teresa Vicenza^4^, Principia Dardano^5^, Bruno Miranda^5^, Pablo V. Escribá^6,7^, Marta Gogliettino^2,†^, Gianna Palmieri^2,8,*,†^

^1^Ampure S.r.l., 80122 Napoli, Italy

^2^Institute of Biosciences and BioResources, National Research Council (IBBR-CNR), 80131 Napoli, Italy

^3^Department of Food Microbiology, Istituto Zooprofilattico Sperimentale del Mezzogiorno, 80055 Portici, Italy

^4^Department of Food Safety, Nutrition and Veterinary Public Health, Istituto Superiore di Sanità, 00161 Roma, Italy

^5^Institute of Applied Sciences and Intelligent Systems, National Research Council (ISASI-CNR), 80131 Napoli, Italy

^6^Laboratory of Molecular Cell Biomedicine, University of the Balearic Islands, 07122 Palma, Spain

^7^Laminar Pharmaceuticals, 07121 Palma, Spain

^8^ Materias S.r.l., 80146 Naples, Italy

*** Correspondence:**

Gianna Palmieri, gianna.palmieri@ibbr.cnr.it

**Supplementary Figure S1. Effect of pH on the secondary and tertiary structure of RiLK3 monitored by spectroscopic techniques**. Far-UV CD spectra of RiLK3 at (**A**) pH 2.0, (**B**) pH 7.0 and (**C**) pH 11.0. Intrinsic fluorescence emission spectra of RiLK3 at (**D**) pH 2.0, (**E**) pH 7.0 and (**F**) pH 11.0. All spectra were recorded at a peptide concentration of 80 µM in buffers at different pHs up to 48 h incubation at 25 °C and in the presence or absence (blue lines) of SDS (50 mM).


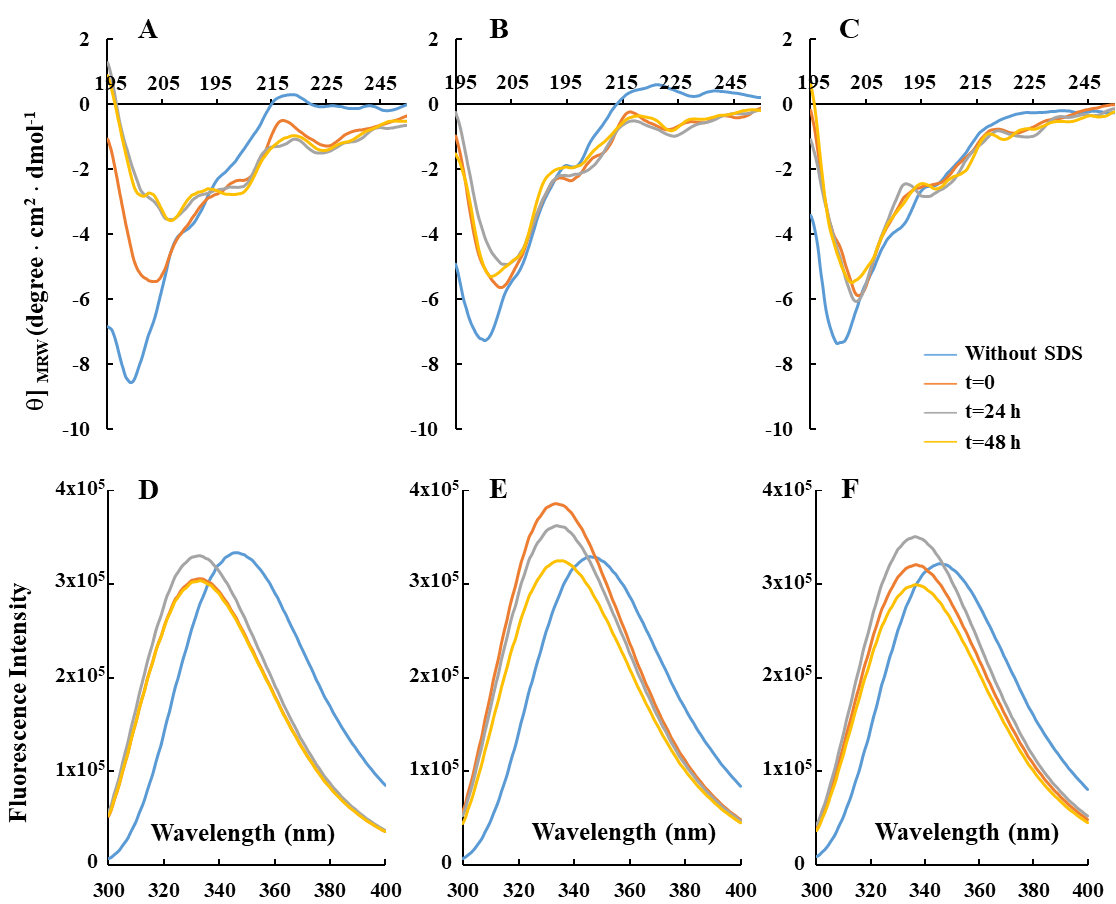


**Supplementary Figure S2. Effect of temperature on the secondary and tertiary structure of RiLK3 monitored by spectroscopic techniques**. Far-UV CD spectra of RiLK3 at (**A**) 4 °C, (**B**) 37 °C and (**C**) 90 °C. Intrinsic fluorescence emission spectra of RiLK3 at (**D**) 4 °C, (**E**) 37 °C and (**F**) 90 °C. All spectra were recorded at a peptide concentration of 80 µM in 10 mM Tris-HCl buffer pH 7.0 in the presence or absence (blue lines) of SDS (50 mM) up to 48 h incubation at 25 °C.


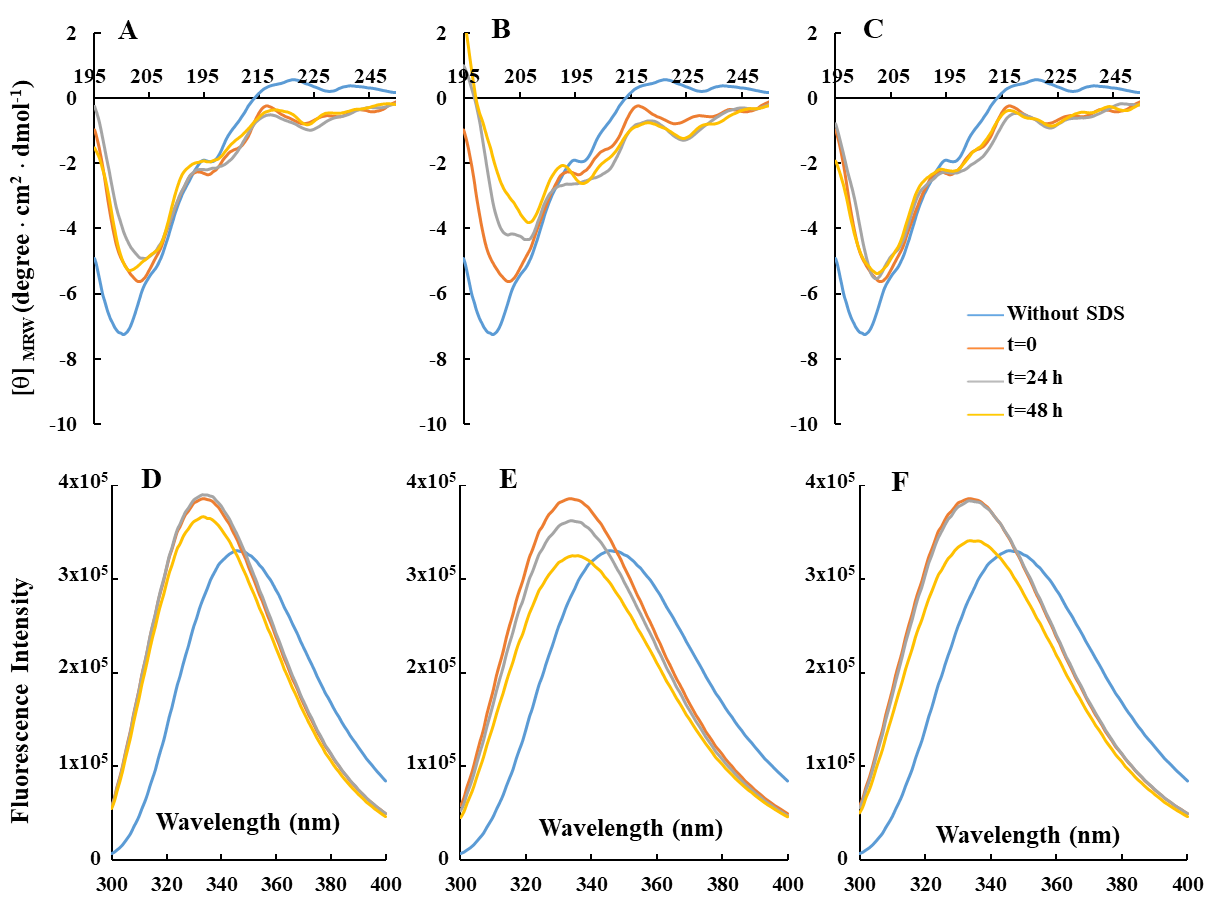


**Supplementary Figure S3. Effect of pH on the secondary and tertiary structure of RiLK1 monitored by spectroscopic techniques**. Far-UV CD spectra of RiLK1 at (**A**) pH 2.0, (**B**) pH 7.0 and (**C**) pH 11.0. Intrinsic fluorescence emission spectra of RiLK1 at (**D**) pH 2.0, (**E**) pH 7.0 and (**F**) pH 11.0. All spectra were recorded at a peptide concentration of 80 µM in buffers at different pHs up to 48 h incubation at 25 °C and in the presence or absence (blue lines) of SDS (50 mM).


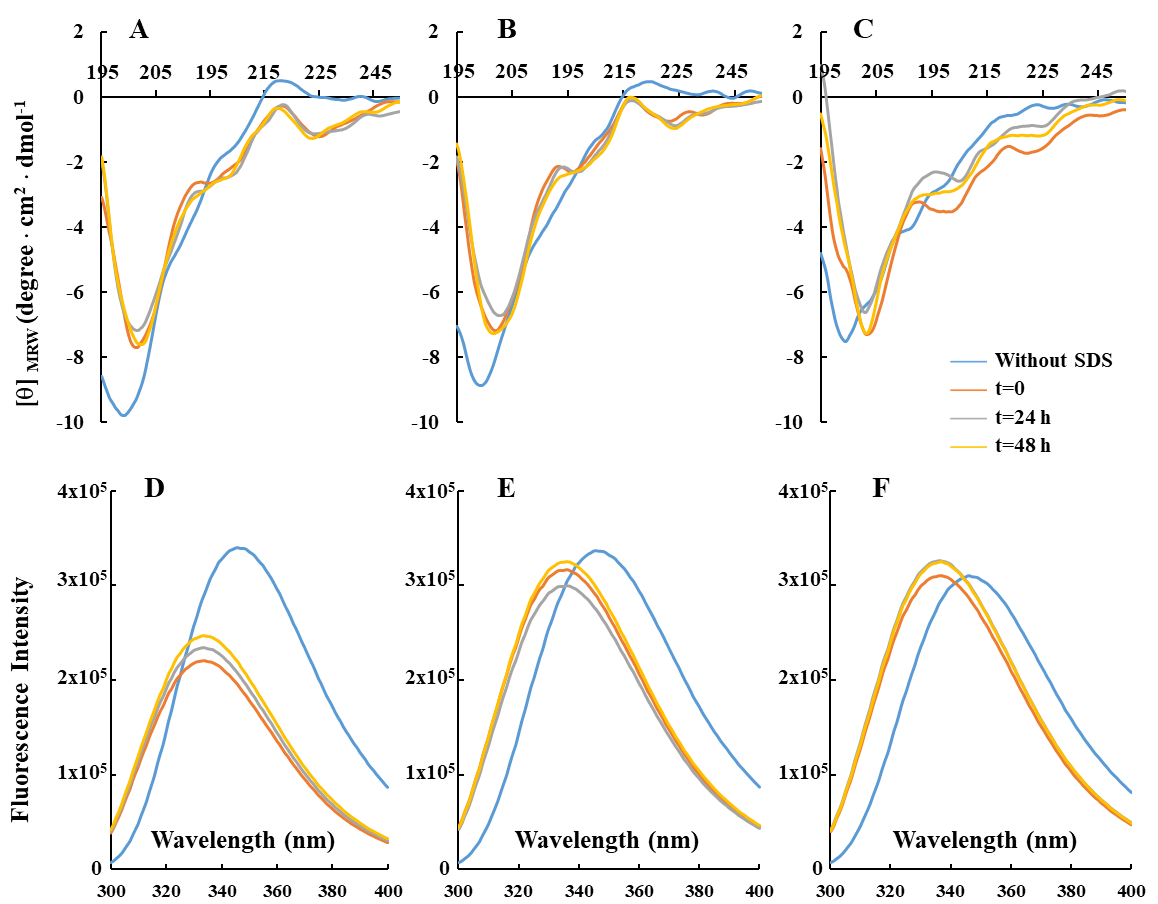


**Supplementary Figure S4. Effect of temperature on the secondary and tertiary structure of RiLK1 monitored by spectroscopic techniques**. Far-UV CD spectra of RiLK1 at (**A**) 4 °C, (**B**) 37 °C and (**C**) 90 °C. Intrinsic fluorescence emission spectra of RiLK1 at (**D**) 4 °C, (**E**) 37 °C and (**F**) 90 °C. All spectra were recorded at a peptide concentration of 80 µM in 10 mM Tris-HCl buffer pH 7.0 in the presence or absence (blue lines) of SDS (50 mM) up to 48 h incubation at 25 °C.


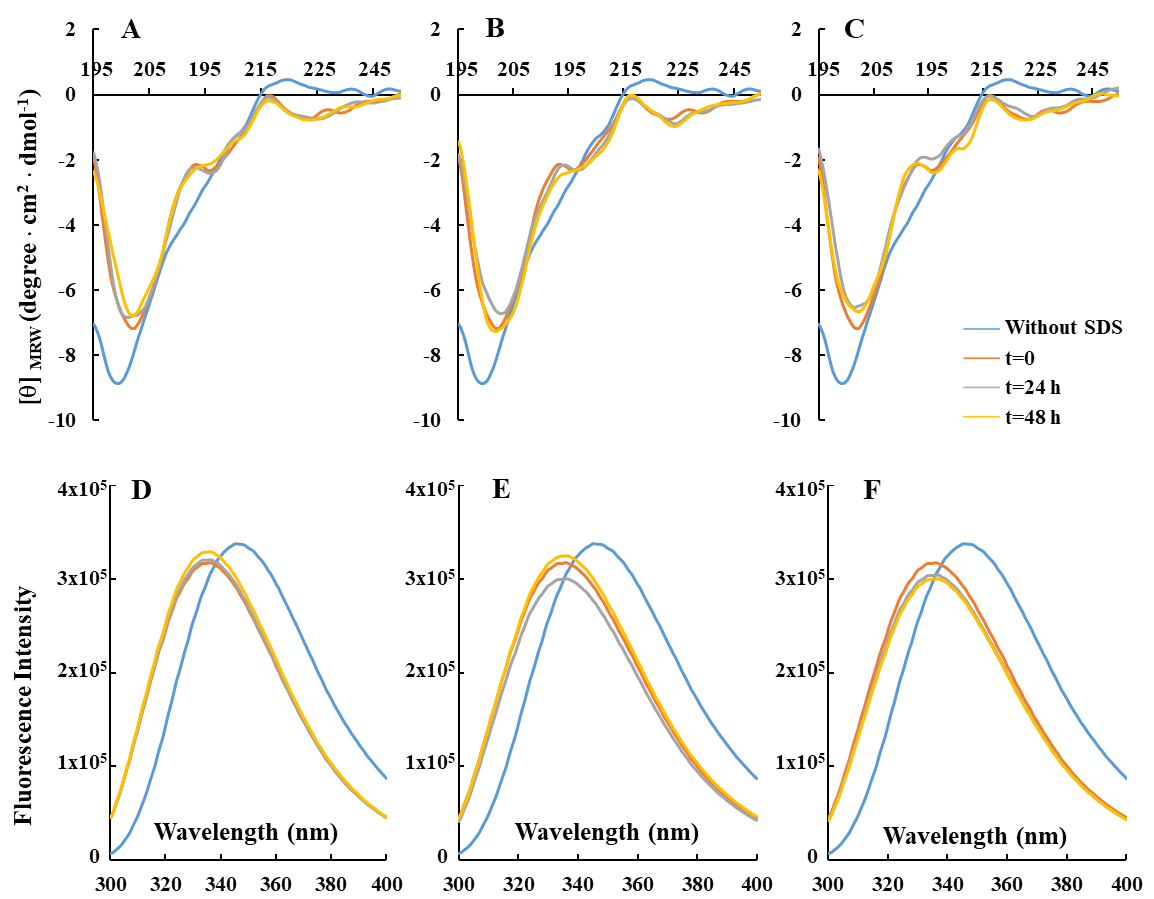


**Supplementary Table S1.** Secondary structure contents of RiLK3 and RiLK1 in the absence or presence of SDS (50 mM) determined by DichroWeb server (K2D method)

|  |  | | **RiLK3** | | | **RiLK1** | | |
| --- | --- | --- | --- | --- | --- | --- | --- | --- |
|  |  | | **Alpha helix**  **(%)** | **Beta-sheet**  **(%)** | **Random coil**  **(%)** | **Alpha helix**  **(%)** | **Beta-sheet**  **(%)** | **Random coil**  **(%)** |
| **WITHOUT SDS** | | | 10 | 39 | 51 | 27 | 32 | 41 |
| **WITH SDS 50 mM** | | **t=0 (min)** | 21 | 27 | 52 | 27 | 32 | 40 |
|  |  | **t=5** | 18 | 29 | 53 | 27 | 33 | 40 |
|  |  | **t=10** | 23 | 29 | 48 | 27 | 32 | 40 |
|  |  | **t=30** | 14 | 31 | 55 | 27 | 32 | 41 |
|  |  | **t=60** | 11 | 34 | 55 | 27 | 32 | 41 |
|  |  | **t=120** | 20 | 31 | 50 | 27 | 32 | 40 |
|  |  | **t=180** | 11 | 35 | 54 | 27 | 32 | 40 |
|  |  | **t=240** | 15 | 33 | 52 | 27 | 32 | 40 |
|  |  | **t=300** | 12 | 37 | 51 | 27 | 32 | 40 |
|  |  | **t=1440** | 23 | 31 | 47 | 27 | 32 | 40 |

**Supplementary Table S2.** Absorption peaks and their tentative assignment for RILK1 and RILK3 peptides

| **RILK1** | | **RILK3** | |  |
| --- | --- | --- | --- | --- |
| **Position (cm^-1^)** | **Absorbance** | **Position (cm^-1^)** | **Absorbance** | **Assignment** |
| - | - | 533 | 0.176 | out of plane C=O bending |
| - | - | 598 | 0.185 | out of plane C=O bending |
| 722 | 0.109 | 721 | 0.201 | OCN bending; out of plane NH bending |
| - | - | 744 | 0.126 | OCN bending; out of plane NH bending |
| 801 | 0.0798 | 801 | 0.122 | out of plane NH bending; C=C stretching |
| 838 | 0.0696 | 838 | 0.1 | C=C stretching |
| - | - | 1048 | 0.0904 | Stretching CO-O-CO |
| 1134 | 0.199 | 1133 | 0.226 | C-O stretching |
| 1183 | 0.202 | 1183 | 0.209 | C-O stretching |
| 1200 | 0.203 | 1200 | 0.213 | C-O stretching |
| - | - | 1361 | 0.0548 | OH bending |
| 1433 | 0.0933 | 1435 | 0.0835 | CH bending |
| 1457 | 0.0876 | 1457 | 0.0797 | CH bending |
| 1472 | 0.079 | - | - | CN stretching; NH bending |
| 1540 | 0.179 | 1538 | 0.152 | CN stretching; NH bending |
| 1622 | 0.351 | 1622 | 0.296 | C=O stretching |
| - | - | 1652 | 0.228 | C=O stretching |
| 1663 | 0.269 | 1660 | 0.229 | C=O stretching |
| 2877 | 0.0831 | - | - | NH stretching |
| 2937 | 0.103 | - | - | NH stretching |
| 2966 | 0.11 | 2965 | 0.0518 | NH stretching |
| 3087 | 0.131 | - | - | NH stretching |
| 3096 | 0.131 | - | - | NH stretching |
| 3107 | 0.129 | - | - | NH stretching |
| 3266 | 0.222 | 3269 | 0.0976 | NH stretching |

**Supplementary Table S3.** Multi-peaks fitting results and their assignment for RILK1

| **RILK1** | | | | | | |  |
| --- | --- | --- | --- | --- | --- | --- | --- |
| **Peak Type** | **Center** | **Height** | **FWHH** | **Area %** | **Attribution** | **tot %** | |
| Lorentzian | 1604 | 0.0453 | 20.569 | 5.6 | side chain |  |  |
| Lorentzian | 1615 | 0.1295 | 20.278 | 15.8 | β-sheet | 52.2 |  |
| Lorentzian | 1623 | 0.1451 | 17.785 | 15.6 | β-sheet |  |  |
| Lorentzian | 1633 | 0.0897 | 15.823 | 8.6 | β-sheet |  |  |
| Lorentzian | 1641 | 0.0403 | 14.979 | 3.6 | β-sheet |  |  |
| Lorentzian | 1647 | 0.0544 | 14.899 | 4.9 | Random | 4.9 |  |
| Lorentzian | 1655 | 0.0669 | 14.936 | 6.0 | α Helix | 5.9 |  |
| Lorentzian | 1661 | 0.0674 | 15.1 | 6.2 | 3_10_ Helix | 6.0 |  |
| Lorentzian | 1667 | 0.0743 | 15.052 | 6.8 | β-Turn | 22.1 |  |
| Lorentzian | 1673 | 0.0697 | 14.966 | 6.3 | β-Turn |  |  |
| Lorentzian | 1679 | 0.0524 | 14.92 | 4.7 | β-Turn |  |  |
| Lorentzian | 1684 | 0.0487 | 14.764 | 4.3 | β-Turn |  |  |
| Lorentzian | 1690 | 0.0509 | 14.724 | 4.5 | β-sheet |  |  |
| Lorentzian | 1695 | 0.0463 | 14.622 | 4.1 | β-sheet |  |  |
| Lorentzian | 1702 | 0.0328 | 14.761 | 2.9 | side chain |  |  |

**Supplementary Table S4.** Multi-peaks fitting results and their assignment for RILK3

| **RILK3** | | | | | | | |
| --- | --- | --- | --- | --- | --- | --- | --- |
| **Peak Type** | **Center** | **Height** | **FWHH** | **Area %** | **Attribution** | **tot %** | |
| Lorentzian | 1602 | 0.012 | 16.245 | 2.5 | side chain |  |  |
| Lorentzian | 1611 | 0.0331 | 15.794 | 6.8 | side chain |  |  |
| Lorentzian | 1619 | 0.0555 | 15.629 | 11.3 | β-sheet | 45.9 |  |
| Lorentzian | 1626 | 0.055 | 15.264 | 10.9 | β-sheet |  |  |
| Lorentzian | 1633 | 0.0493 | 14.884 | 9.6 | β-sheet |  |  |
| Lorentzian | 1641 | 0.0342 | 14.737 | 6.6 | β-sheet |  |  |
| Lorentzian | 1647 | 0.0288 | 14.792 | 5.5 | Random | 11.2 |  |
| Lorentzian | 1653 | 0.0309 | 14.712 | 5.9 | Random |  |  |
| Lorentzian | 1659 | 0.04 | 14.633 | 7.6 | α Helix | 7.6 |  |
| Lorentzian | 1666 | 0.0413 | 14.598 | 7.8 | β-Turn | 23.4 |  |
| Lorentzian | 1673 | 0.0354 | 14.654 | 6.7 | β-Turn |  |  |
| Lorentzian | 1679 | 0.0244 | 14.755 | 4.7 | β-Turn |  |  |
| Lorentzian | 1683 | 0.0218 | 14.516 | 4.1 | β-Turn |  |  |
| Lorentzian | 1690 | 0.0244 | 14.427 | 4.6 | β-sheet |  |  |
| Lorentzian | 1695 | 0.0159 | 14.438 | 3.0 | β-sheet |  |  |
| Lorentzian | 1702 | 0.0121 | 14.449 | 2.3 | side chain |  |  |
